# Polypharmacy in atrial fibrillation: A prospective analysis of mortality and ischemic stroke using the Clinical Practice Research Datalink

**DOI:** 10.1002/joa3.12961

**Published:** 2023-11-27

**Authors:** Natasha Slater, Simon White, Martin Frisher

**Affiliations:** ^1^ School of Pharmacy and Bioengineering Keele University Staffordshire UK

**Keywords:** atrial fibrillation, CPRD, ischemic stroke, mortality, polypharmacy

## Abstract

**Background:**

Observational studies of polypharmacy and the risk of death or stroke in individuals with atrial fibrillation (AF) have produced inconsistent findings. By using propensity score matching (PSM) and Cox regression, this study aimed to determine whether polypharmacy (five to nine medicines) in the 3 months following AF diagnosis, is associated with an increased risk of death or ischemic stroke, compared to non‐polypharmacy (one to four medicines).

**Methods:**

A prospective cohort study using data from the Clinical Practice Research Datalink (2006–2019). Data from 23 629 individuals with AF were analyzed. Cox regression models were adjusted for age, gender, morbidities, obesity, alcohol, smoking, and wealth. In the PSM models, cases and controls with near identical health profiles were selected from the study pool. The risk of death and stroke were presented as hazard ratios (HRs) with 95% confidence intervals (CIs).

**Results:**

68.9% (*n* = 16 271) of the participants had polypharmacy. PSM showed that polypharmacy was associated with an increased risk of death during follow‐up (HR 1.32; 95% CI: 1.19–1.47, *p* < .01), but not ischemic stroke (HR 0.84; 95% CI: 0.69–1.02, *p* = .08).

**Conclusion:**

Polypharmacy was associated with an increased risk of death during follow‐up, but not ischemic stroke, in individuals with AF. The effects of comorbidity and other confounding factors were reduced by using PSM. This study focused on the overall medication burden; however, further research is needed to identify which specific medications in polypharmacy regimens increase the risk of mortality in AF. These findings could inform prescribing practices in the future.

## Graphical abstract

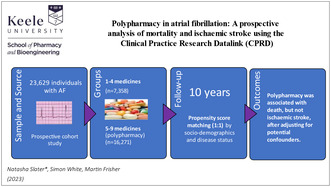

## 1 INTRODUCTION

Polypharmacy, the concomitant use of multiple medications by one individual, has increased over the past decade, because of a growing aging population and rising numbers of individuals with chronic conditions and multi‐morbidities.^1^, ^2^, ^3^ Polypharmacy has been previously shown to be associated with adverse outcomes, including adverse drug reactions, hospitalizations, falls, and mortality.^4^

Despite the rising prevalence of polypharmacy, there are limited data regarding polypharmacy in individuals with atrial fibrillation (AF). AF is the most common sustained cardiac arrhythmia in England, affecting approximately 1.4 million adults.^5^ The cause of AF is not fully understood; however, multiple drug classes are used to manage the condition. Furthermore, AF commonly co‐exists with other conditions, including hypertension, atherosclerosis, and diabetes, thus increasing the likelihood that individuals with AF will experience polypharmacy.^6^ AF is also an independent risk factor for adverse outcomes, including ischemic stroke, chronic heart failure, and other cardiovascular (CV)‐related complications.^5^

Previous studies which have examined the associations between polypharmacy and adverse outcomes, in AF, have been post‐hoc analyses of trial data.^7^, ^8^, ^9^ Trial inclusion criteria, varying levels of statistical adjustments and varying definitions of polypharmacy make it difficult to compare these studies, and mixed findings have been reported.^7^, ^8^, ^9^

Data from the Clinical Practice Research Datalink (CPRD) were analyzed in this study. By using propensity score matching (PSM) and Cox regression, we aimed to determine whether polypharmacy (five to nine prescribed medicines) in the 3 months following AF diagnosis, is associated with an increased risk of death or ischemic stroke, compared to non‐polypharmacy (one to four prescribed medicines).

## 2 METHODS

### 2.1 Sample and participants

The CPRD gathers anonymous patient data from over 1800 GP surgeries, located within the United Kingdom, on a monthly basis.^10^ CPRD data have been shown to be broadly representative of the UK general population, in terms of age, gender, and ethnicity, previously.^11^, ^12^ CPRD data are stored in two datasets, CPRD GOLD and CPRD Aurum, which collectively “encompass 16 482 304 patients who are currently registered as research‐acceptable patients.”^11^, ^13^ This prospective cohort study analyzed data recorded in the CPRD dataset, CPRD GOLD, between June 1, 2006 and April 4, 2019.

Data were extracted from the following six files of the CPRD GOLD dataset in this study: Patient, Practice, Clinical, Additional Clinical Details, Test, and Therapy.^14^ The Patient file provided demographic data, surgery registration data, transfer out dates and transfer out reasons, death dates, and information about whether each patient record was “up‐to‐standard”, according to the CPRD quality checks.^14^ Further “up‐to‐standard” data were available in the Practice file, along with information about the location of the participating GP surgeries. The Clinical file provided clinical event data, including the date of AF diagnosis (index date). Further data about each clinical event were available from the Additional Clinical Details file. Blood test data and anthropometric measurement data were available in the Test file. Finally, prescribed medication data were available in the Therapy file.^14^ The CPRD GOLD dataset was linked to the patient‐level Index of Multiple Deprivation (IMD) 2015 dataset.^10^ Data were extracted using Read code lists. The Read code lists for AF diagnoses, ischemic stroke, and all prognostic factors were reviewed and approved by two academic General Practitioners, and the Independent Scientific Advisory Committee (ISAC) prior to study approval and data extraction. All Read code lists are available as online supplementary material (Tables S1–S12).

### 2.2 Ethical approval

The CPRD has been granted a favorable ethical opinion by the Health Research Authority, to provide anonymized patient data for use in observational research (reference number 05/MRE04/87).^10^ Approval was also granted by the ISAC (Protocol 18_151). Additional ethical review was not required for this study.

### 2.3 Inclusion criteria

Eligible participants must have had an AF diagnosis, recorded in the Clinical file of CPRD GOLD, between June 1, 2006 and April 4, 2019. This study did not differentiate between incident and prevalent cases of AF diagnoses, instead it focused on whether participants had an AF diagnosis recorded within the study period. The participant must also have been prescribed a minimum of one medicine in the first 3 months following their AF diagnosis. Participants who were not prescribed medications within the 3 months of AF diagnosis were excluded, as prescribed medications were the exposure in this study. Previous feasibility counts using CPRD data showed that the number of participants prescribed no medications after an AF diagnosis was 28%, (*n* = 825/2916). Finally, the participant must have “acceptable data” according to the CPRD quality standards, and have been registered with an “up‐to‐standard” GP surgery, for a minimum of 2 years prior to their AF diagnosis.^15^ These quality standards ensured that only “research‐quality patients and periods of quality data recording” were extracted for the analyses.^11^

### 2.4 Study exposures and outcomes

The index date for each participant was the date of the earliest AF diagnosis, recorded in the Clinical file of CPRD GOLD, between June 1, 2006 and April 4, 2019. Follow‐up commenced 3 months after the index date. The maximum follow‐up period for this study was 10 years. The follow‐up period was terminated early if any of the following events occurred: the incidence of death, the incidence of ischemic stroke, or the patient transferred out of the practice. Any participant who experienced an outcome of interest or transferred out of the practice in the 3 months after index date, but before follow‐up commenced, were excluded from the analyses. However, previous feasibility counts using CPRD data showed that the immortal time bias risk was low (<1%), because of the short time period (3 months) between the index date and the start of follow‐up.

The number of different prescribed medications in the 3 months following AF diagnosis was the exposure in this study. Prescribed medication data were obtained from the Therapy file in CPRD GOLD, and the extracted data were linked to a Product dictionary. The latter provided the following information: product names (generic or proprietary), product codes, drug substances (active ingredients), strengths, formulations, routes of administration, British National Formulary (BNF) Chapters and BNF codes.^15^ For each participant, the number of prescribed medications in the 3 months following AF diagnosis, was determined by conducting a count of drug substances. The reason for selecting drug substance, rather than product code, was to ensure that appliances, for example insulin pen needles and catheters were excluded from the prescribed medication count.

Polypharmacy was defined as five to nine prescribed medications in the 3 months following AF diagnosis, while non‐polypharmacy was defined as one to four prescribed medications in the 3 months following AF diagnosis. These definitions have been used in other studies previously.^3^

The incidence of death (defined as a death date ≥ study index date and documented in the Patient file), and the incidence of ischemic stroke (defined as a record of a Read code for ischemic stroke, and documented in the Clinical file, with an event date ≥ study index date), recorded between June 1, 2006 and 4th April 4, 2019, were the primary outcomes for this study.

### 2.5 Prognostic factors

The Clinical file, Test file, and Additional Clinical Details file in CPRD GOLD, in addition to the English IMD linked dataset, were accessed to obtain the prognostic factor data for this study. Prognostic factor data recorded between the index date and 2 years prior to the index date were extracted and included in the statistical analyses. Previous studies which have examined the adverse outcomes associated with polypharmacy, in AF, have used fewer prognostic factors in their analyses, compared to this study.^7^, ^8^, ^9^

The following pre‐existing medical conditions were included as prognostic factors in this study: chronic obstructive pulmonary disease, diabetes mellitus, heart failure, hypertension, ischemic heart disease and other ischemic CV conditions, peripheral vascular disease, previous ischemic stroke, previous myocardial infarction, obstructive sleep apnea, and thyroid disorders. These medical conditions were defined as Read codes in the participant's Clinical file. The look back period for these confounders was 2 years prior to the index date. For each of these medical conditions, the participant's disease status was recorded using a binary categorical variable.

These medical conditions have been shown to be independently associated with ischemic strokes and mortality previously, and the risk is enhanced in individuals who have AF as a comorbidity. For example, the mortality rate in individuals with AF and heart failure is doubled, compared to individuals with AF alone.^16^, ^17^, ^18^, ^19^, ^20^, ^21^, ^22^

Renal insufficiency, obesity, smoking, alcohol consumption, and wealth were also included as prognostic factors. If more than one record was available, the latest (i.e., the most recent) record was selected. Renal insufficiency was defined as a record of estimated glomerular filtration rate ≤30 mL/min/1.73 m^2^ in the participant's Test file. The co‐existence of AF and renal insufficiency has been shown to be associated with an increased risk of thromboembolism,^23^ and mortality previously.^24^, ^25^, ^26^

Obesity was defined as a body mass index (BMI) ≥30 kg/m^2^, recorded in the participant's Clinical file. Smoking status was recorded as non‐smoker or smoker, while alcohol consumption status was recorded as non‐drinker, drinker, or ex‐drinker. Our previous research found a significant association between obesity and polypharmacy prevalence in older people.^27^ Furthermore, increasing BMI is associated with adverse outcomes, including mortality and ischemic stroke.^29^ Similarly, the associations between smoking and regular alcohol consumption, with mortality and ischemic stroke are well established.^30^, ^31^, ^32^

Finally, wealth data were extracted from the English IMD 2015 linked dataset, and participants were allocated to one of five wealth quintiles. Quintiles were determined according to deprivation scores, at “lower‐layer super output area” level.^33^ A deprivation score is a composite score which takes into account the following levels of deprivation: income, employment, health and disability, education and training, barriers to housing or services, crime, and the living environment.^33^, ^34^ Therefore, quintile 1 was the wealthiest (i.e., lowest deprivation scores), while quintile 5 was the poorest (i.e., highest deprivation scores). IMD data were not available for all study participants, so rather than excluding these participants from the main analyses, their wealth data were coded as “missing” and included as a separate category in the models.

### 2.6 Data analysis

Descriptive statistics and chi‐square tests of independence were used initially to profile each group according to participant demographics, and to examine the association between polypharmacy and study outcomes (death and ischemic stroke). Following this, Cox proportional hazards models were used, and hazard ratios (HRs) with 95% confidence intervals (CIs) were calculated for the risk of death and ischemic stroke. The models were adjusted for the age, gender, 11 diagnosed conditions, obesity, alcohol consumption, smoking, and wealth. The minimum sample size required for adjusted models was 354.^35^ Results were considered to be statistically significant if *p* < .05. Missing data were coded as “missing” and were included (but not reported) as a separate category in all the models.

This study also used optimal PSM without replacement, with a caliper width of 0.1. Participants in the polypharmacy group were matched (1:1) to participants with near identical health profiles in the non‐polypharmacy group by age, gender, 11 diagnosed conditions, obesity, alcohol consumption, smoking, and wealth (Tables S1–S12). This approach reduced confounding, as the only measured difference between the matched groups was the number of medications prescribed in the 3 months following AF diagnosis. The relative multivariate imbalance before matching was 0.541, which reduced to 0.371 after matching. All analyses were undertaken using IBM Statistical Package for Social Sciences (V.26.0).

## 3 RESULTS

### 3.1 Participant characteristics

There were 23 629 participants eligible for inclusion in this study. In the 3 months following AF diagnosis, 68.9% (*n* = 16 271) of the participants were prescribed between five and nine medicines concurrently (polypharmacy), while 31.1% (*n* = 7, 358) were prescribed between one and four medicines concurrently (non‐polypharmacy), as shown in Table 1. Participants with polypharmacy were older (mean age 75 years) compared to participants with non‐polypharmacy (mean age 69 years; Table 1). There were also more women in the polypharmacy group, compared to the non‐polypharmacy group (Table 1).

**TABLE 1:** Participant characteristics at study entry (*n* = 23 629).

	One to four medicines (non‐polypharmacy)	Five to nine medicines (polypharmacy)	Sig.
Total (*n* = 23 629)	7358	16 271	
Age			
Age (mean ± SD)	69 ± 14	75 ± 11	
Age (18–30 years) *n* (%)	59 (0.8%)	17 (0.1%)	*p* < .01
Age (31–50 years) *n* (%)	700 (9.5%)	372 (2.3%)	
Age (51–70 years) *n* (%)	2921(39.7%)	4628 (28.4%)	
Age (71–84 years) *n* (%)	2736 (37.2%)	8157 (50.1%)	
Age (≥85 years) *n* (%)	942 (12.8%)	3097 (19.0%)	
Gender			
Female *n* (%)	2852 (38.8%)	7748 (47.6%)	*p* < .01
Diagnosed conditions in the 2 years prior to AF diagnosis			
COPD *n* (%)	153 (2.1%)	848 (5.2%)	*p* < .01
Diabetes *n* (%)	244 (3.3%)	1672 (10.3%)	*p* < .01
Heart failure *n* (%)	116 (1.6%)	1058 (6.5%)	*p* < .01
Hypertension *n* (%)	660 (9.0%)	1798 (11.1%)	*p* < .01
IHD and other ischemic cardiovascular conditions *n* (%)	155 (2.1%)	1031 (6.3%)	*p* < .01
Myocardial infarction *n* (%)	28 (0.4%)	297 (1.8%)	*p* < .01
Previous ischemic stroke *n* (%)	311 (4.2%)	1077 (6.6%)	*p* < .01
Peripheral vascular disease *n* (%)	52 (0.7%)	218 (1.3%)	*p* < .01
Sleep apnea *n* (%)	47 (0.6%)	183 (1.1%)	*p* < .01
Thyroid disorders *n* (%)	89 (1.2%)	275 (1.7%)	*p* < .01
Poor renal function (eGFR ≤30 mL/min/1.73 m^2^) *n* (%)	28 (0.4%)	143 (0.9%)	*p* < .01
Lifestyle factors at study entry			
Non‐obese (BMI ≤30 kg/m^2^) *n* (%)	2598 (35.3%)	6891 (42.4%)	*p* < .01
Obese (BMI ≥30 kg/m^2^) *n* (%)	1025 (13.9%)	3437 (21.1%)	
Non‐drinker (alcohol) *n* (%)	407 (5.5%)	1555 (9.6%)	*p* < .01
Drinker (alcohol) *n* (%)	2255 (30.6%)	5651 (34.7%)	
Ex‐drinker (alcohol) *n* (%)	70 (1.0%)	224 (1.4%)	
Non‐smoker *n* (%)	2797 (38.0%)	6351 (39.0%)	*p* < .01
Smoker *n* (%)	665 (9.0%)	1423 (8.7%)	
Wealth at study entry			
Wealth quintile 1 (wealthiest) *n* (%)	1219 (16.6%)	2227 (13.7%)	*p* < .01
Wealth quintile 2 *n* (%)	1034 (14.1%)	2168 (13.3%)	
Wealth quintile 3 *n* (%)	891 (12.1%)	2006 (12.3%)	
Wealth quintile 4 *n* (%)	652 (8.9%)	1560 (9.6%)	
Wealth quintile 5 (poorest) *n* (%)	406 (5.5%)	1226 (7.5%)	

Participants with polypharmacy had more diagnosed conditions in the 2 years prior to AF diagnosis, compared to participants in the non‐polypharmacy group. The most commonly diagnosed conditions among participants with polypharmacy were hypertension (11.1%), diabetes mellitus (10.3%), and heart failure (6.5%). Hypertension (9.0%) and diabetes mellitus (3.3%) were also the most prevalent diagnosed conditions in the non‐polypharmacy group (Table 1).

There were similar proportions of individuals who smoked and consumed alcohol in each group (Table 1). However, there were more obese (BMI ≥30 kg/m^2^) participants in the polypharmacy group (21.1%), compared to the non‐polypharmacy group (13.9%) (Table 1). Finally, in comparison to participants in the non‐polypharmacy group, fewer participants in the polypharmacy group were in wealth quintile 1 (wealthiest) (13.7%), and more participants with polypharmacy were in wealth quintile 5 (poorest) (7.5%) (Table 1).

Prescribed medication data were also examined. Overall, 134 745 medications were prescribed for the study participants (*n* = 23 629) in the 3 months following AF diagnosis. Prescribed medication data were stratified by polypharmacy group at baseline and BNF Chapters, and the results are available as supplementary material (Table S13). However, in contrast to the data presented in the main paper, the data in these tables represent the number of medications prescribed, rather than the number of study participants. The rationale for this approach with the medication data was to examine prescribing at group level (i.e., polypharmacy vs. non‐polypharmacy), rather than at individual participant level. BNF Chapter 2 medication data were further stratified into drug class and are available in Table S14.

### 3.2 The associations between polypharmacy in the 3 months following AF diagnosis, and study outcomes (death and ischemic stroke): An initial examination

**FIGURE 1:**
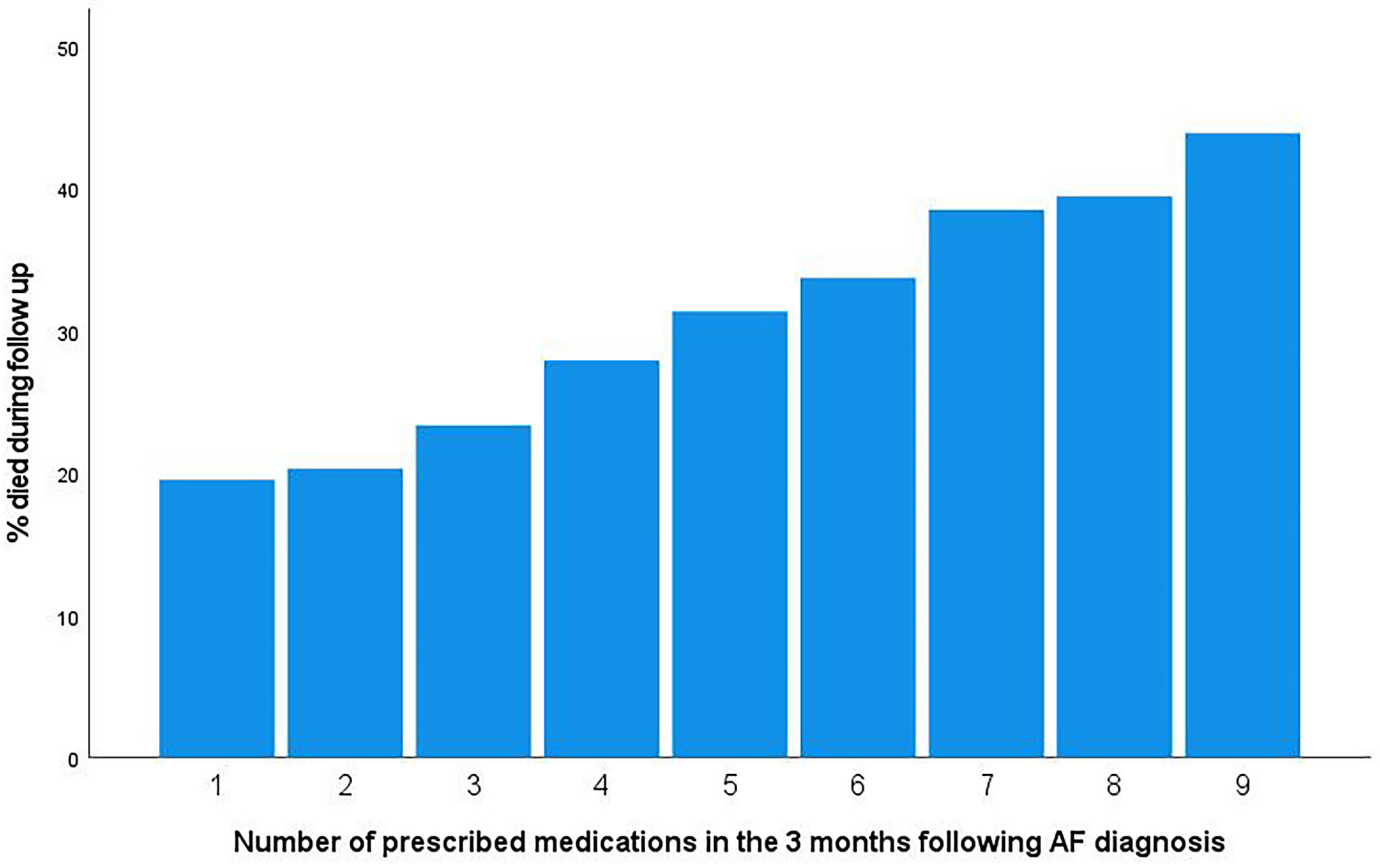
Number of prescribed medications in relation to mortality.

Before the end of the study, 33.1% (*n* = 7816/23 629) of the participants had died, and 8.9% (*n* = 2106/23 629) had experienced an ischemic stroke. Outcome data were stratified by polypharmacy group at baseline. There were more deaths in the polypharmacy group (37.2%), compared to the non‐polypharmacy group (24.0%) (Data S1; Figure 1). However, the percentages of ischemic strokes during follow‐up were similar for both groups (9.0% for the non‐polypharmacy group and 8.9% for the polypharmacy group, respectively). The mean duration of follow‐up varied between the study groups (5.5 years ±3 for the non‐polypharmacy group and 5.0 years ±3 for the polypharmacy group).

### 3.3 The associations between polypharmacy in the 3 months following AF diagnosis, and study outcomes (death and ischemic stroke): Adjusted analyses

Cox regression showed that polypharmacy, in the first 3 months following AF diagnosis, was associated with an increased risk of death during follow‐up (unadjusted HR 1.73; 95% CI; 1.64 to 1.82, *p* < .01; Table 2). After adjusting for participant age, the HR reduced to 1.31 (95% CI: 1.24 to 1.38, *p* < .01; Table 2). The HR reduced further to 1.26 (95% CI: 1.20 to 1.34, *p* < .01) when the model was adjusted for diagnosed conditions, lifestyle factors, and wealth, in addition to age and gender (Table 2).

**TABLE 2:** Cox regression to examine the association between polypharmacy and study outcomes during follow‐up (*n* = 23 629).

	Death hazard ratio (95% CI)	Ischemic stroke hazard ratio (95% CI)
Polypharmacy	1.73 (1.64–1.82, *p* < .01)	1.10 (0.99–1.20, *p* = .05)
Polypharmacy: adjusted for gender	1.72 (1.63–1.81, *p* < .01)	1.08 (0.98–1.18, *p* = .12)
Polypharmacy: adjusted for age	1.31 (1.24–1.38, *p* < .01)	0.93 (0.84–1.02, *p* = .10)
Polypharmacy: adjusted for age, gender, and diagnosed conditions	1.26 (1.19–1.33, *p* < .01)	0.89 (0.81–0.98, *p* = .02)
Polypharmacy: adjusted for age, gender, diagnosed conditions, and lifestyle factors	1.26 (1.20–1.34, *p* < .01)	0.90 (0.81–0.98, *p* = .03)
Polypharmacy: adjusted for age, gender, diagnosed conditions, lifestyle factors, and wealth	1.26 (1.20–1.34, *p* < .01)	0.90 (0.81–0.98, *p* = .03)

Cox regression was also used to examine the association between polypharmacy and ischemic stroke. The unadjusted results showed that polypharmacy, in the first 3 months following AF diagnosis, was associated with an increased risk of ischemic stroke during follow‐up (unadjusted HR 1.10, 95% CI: 1.00 to 1.20, *p* < .05; Table 2). However, when the models were adjusted in a stepwise manner, for gender, age, diagnosed conditions, lifestyle factors, and wealth, the HR reduced to 0.90 (95% CI: 0.81 to 0.98, *p* = .03; Table 2).

### 3.4 The associations between polypharmacy in the 3 months following AF diagnosis, and study outcomes (death and ischemic stroke): Propensity score matched

Overall, 2451 participants in the polypharmacy group were propensity score matched (1:1) by age, gender, diagnosed conditions in the 2 years prior to AF diagnosis, lifestyle factors, and wealth, to 2451 participants in the non‐polypharmacy group. Demographic data for the propensity score matched groups are presented in Table 3.

**TABLE 3:** Demographic data for the propensity score matched groups (non‐polypharmacy and polypharmacy; *n* = 4902).

	One to four medicines (matched non‐polypharmacy)	Five to nine medicines (matched polypharmacy)
Total (*n*)	2451	2451
Age (18–30 years) *n* (%)	0 (0.0%)	0 (0.0%)
Age (31–50 years) *n* (%)	60 (2.4%)	60 (2.4%)
Age (51–70 years) *n* (%)	877 (35.8%)	877 (35.8%)
Age (71–84 years) *n* (%)	1204 (49.1%)	1204 (49.1%)
Age (≥85 years) *n* (%)	310 (12.6%)	310 (12.6%)
Female *n* (%)	1019 (41.6%)	1019 (41.6%)
Diagnosed conditions at study entry		
COPD *n* (%)	9 (0.4%)	9 (0.4%)
Diabetes *n* (%)	32 (1.3%)	32 (1.3%)
Heart failure *n* (%)	5 (0.2%)	5 (0.2%)
Hypertension *n* (%)	51 (2.1%)	51 (2.1%)
IHD or other cardiovascular conditions *n* (%)	3 (0.1%)	3 (0.1%)
Myocardial infarction *n* (%)	0 (0.0%)	0 (0.0%)
Previous stroke *n* (%)	19 (0.8%)	19 (0.8%)
Peripheral vascular disease *n* (%)	1 (0.0%)	1 (0.0%)
Sleep apnea *n* (%)	0 (0.0%)	0 (0.0%)
Thyroid disorders *n* (%)	1 (0.0%)	1 (0.0%)
Poor renal function (eGFR ≤30 mL/min/1.73 m^2^) *n* (%)	0 (0.0%)	0 (0.0%)
Lifestyle factors at study entry		
Obese (BMI ≥30 kg/m^2^) *n* (%)	220 (9.0%)	220 (9.0%)
Non‐obese (BMI ≤30 kg/m^2^) *n* (%)	813 (33.2%)	813 (33.2%)
Non‐drinker (alcohol) *n* (%)	64 (2.6%)	64 (2.6%)
Drinker (alcohol) *n* (%)	692 (28.2%)	692 (28.2%)
Ex‐drinker (alcohol) *n* (%)	2 (0.1%)	2 (0.1%)
Non‐smoker *n* (%)	887 (36.2%)	887 (36.2%)
Smoker *n* (%)	102 (4.2%)	102 (4.2%)
Wealth at study entry		
Wealth quintile 1 (wealthiest) *n* (%)	387 (15.8%)	387 (15.8%)
Wealth quintile 2 *n* (%)	278 (11.3%)	278 (11.3%)
Wealth quintile 3 *n* (%)	256 (10.4%)	256 (10.4%)
Wealth quintile 4 *n* (%)	126 (5.1%)	126 (5.1%)
Wealth quintile 5 (poorest) *n* (%)	60 (2.4%)	60 (2.4%)

Abbreviations: COPD, chronic obstructive pulmonary disease; eGFR, estimated glomerular filtration rate.

The prescribed medication data for the propensity score matched groups are available in Table S15. There were 7287 medications prescribed for the participants in the non‐polypharmacy group, while there were 16 567 medications prescribed for the participants in the polypharmacy group, in the 3 months following AF diagnosis. Prescribed medication data for the matched group were stratified by BNF chapters. Prescribing in the matched non‐polypharmacy group was less diverse, compared to the matched polypharmacy group; however, cardiovascular medicines (BNF Chapter 2), central nervous system medicines (BNF Chapter 4), and gastrointestinal medicines (BNF Chapter 1) were most commonly prescribed types of medications in both groups.

In the propensity score matched groups, 31.9% of the polypharmacy group died during follow‐up, compared to 25.5% in the non‐polypharmacy group. The percentage of ischemic strokes also differed between the propensity score matched group, with 7.4% of participants in the polypharmacy and 9.2% of participants in the non‐polypharmacy group experiencing an ischemic stroke during follow‐up. Cox regression was used to examine the associations between polypharmacy in the 3 months following AF diagnosis, and study outcomes (death and ischemic stroke) during follow‐up, in the propensity score matched groups. Results showed that polypharmacy was associated with an increased risk of death (HR 1.32; 95% CI 1.19 to 1.47, *p* < .01) during follow‐up, but not ischemic stroke (HR 0.84; 95% CI 0.69 to 1.02, *p* = .08).

## 4 DISCUSSION

The propensity score matched results showed that polypharmacy, in the first 3 months following AF diagnosis, was associated with an increased risk of death during follow‐up, but there was no significant association between polypharmacy and ischemic stroke. These findings were similar to the adjusted Cox regression results.

Mixed findings have been reported regarding the associations between polypharmacy and study outcomes (death and ischemic stroke) previously. Piccini et al.^8^ and Eggebrecht et al. supported the findings from this study; however, Focks et al.^7^ reported that polypharmacy was associated with death and ischemic stroke during follow‐up.

There may be several explanations for the differing ischemic stroke findings reported. First, Focks et al.^7^ adjusted their models for age, gender, and country of origin only, whereas Piccini et al.^8^ Eggebrecht et al.^33^ and the current study, adjusted the models for a greater number of potential confounders, including diagnosed conditions. Focks et al.^7^ acknowledged that although they reported an association between polypharmacy and ischemic stroke, it is possible that the association may have diminished if their analyses had been adjusted for “comorbidities at baseline.” Second, this is the first study to examine the associations between polypharmacy, death, and ischemic stroke, in individuals with AF, by analyzing primary care data. Previous studies have examined the associations by conducting post‐hoc analyses of trial data.^7^, ^8^, ^9^ The latter analyses may be limited by the data collected during the trial. For example, Proietti et al.^9^ analyzed cardiovascular medication data only, in relation to polypharmacy and adverse outcomes, as data regarding other types of medications were not collected from participants at trial enrollment. The trial inclusion criteria may have also influenced the results reported. In the current study, data from all participants with AF were analyzed, irrespective of their thromboembolic risk, thus producing more generalizable findings. However, findings from the previous post‐hoc analyses may not be generalizable, as Piccini et al.^8^ analyzed polypharmacy data from participants who had a “moderate to high risk of a stroke” only, while Focks et al.^7^ analyzed data from participants with AF, who had at least one additional risk factor for thromboembolism. Finally, different numeric thresholds were used to define polypharmacy in these studies, which may have impacted the results reported.^7^, ^8^

Our previous research into the composition of polypharmacy regimens showed that cardiovascular medicines are most commonly taken by participants with polypharmacy.^35^ This study focused on the overall burden of medication, so further research at drug class or individual drug level is required to develop our understanding regarding the lack of association between polypharmacy and ischemic stroke, in AF.

The appropriateness of prescribing was not examined in our study; however, if people with AF and polypharmacy have an increased risk of mortality, then it may be prudent for prescribers to consider deprescribing strategies once the appropriateness of each medication in the regimen has been considered, on a case‐by‐case basis. If these medications are deprescribed with the intention of reducing the overall medication burden only, without considering medication appropriateness, then the resultant effect could be worsening disease management.

This study also confirms our previous research into the factors associated with polypharmacy, by showing that lower wealth and obesity were significantly associated with polypharmacy prevalence, in addition to increasing age and morbidities.^27^

This research has some key strengths. Data from 23 629 individuals with AF were analyzed. Moreover, this is the first study to examine the association between polypharmacy, death, and ischemic stroke, in individuals with AF, by analyzing primary care data. This is also the first study to examine the associations using PSM. Previous studies have used regression models, with varying levels of adjustment,^7^, ^8^, ^9^ whereas PSM ensures that all study participants are similar at study entry.

Although this study has a number of strengths, there are some limitations. Only prescribed medication data are available in the CPRD GOLD dataset; therefore, it is possible that the prevalence of polypharmacy may have been underestimated in this study, as medications which can be purchased over‐the‐counter from pharmacies were not included in the count. It was also not possible to determine medication adherence from the dataset, so an assumption was made that all prescribed medications were taken by the study participants, according to the prescriber's directions. Another limitation is that participants were allocated into groups, according to the number of medications they were prescribed in the 3 months following AF diagnosis, and hence it was assumed that their polypharmacy status did not change during the study period. This study included a greater number of confounders in the analyses, compared to previous studies; however, the look back period for these confounders was relatively short (2 years). PSM and adjusted Cox regression were used in this study to reduce confounding; however, as with all observational studies, there may be other factors which have not been accounted for in the analyses, which could explain the observed associations between polypharmacy and the study outcomes, in AF, for example disease severity or medication adherence. The cause of death data were not available for these analyses, and thus may have provided further insight into the associations found. Finally, there was a small proportion of participants in the matched sample, relative to the main sample, which was because of the PSM algorithm used in the analyses.

## 5 CONCLUSION

Polypharmacy was associated with an increased risk of mortality during follow‐up, but not ischemic stroke, in the 3 months following AF diagnosis. Furthermore, this study reduced the confounding effect of morbidity, as the underlying cause of the association, by using PSM.

This study focused on the overall medication burden; however, future research conducted at drug class or individual drug level, could identify which medications, or combinations of medications, within polypharmacy regimens are associated with an increased risk of death. Identifying these medications could help to inform prescribing decisions and deprescribing practices in AF and hence this study provides baseline data for the future research. Details about the cause of death would also provide a valuable insight into the associations reported, particularly as mortality is often highly confounded. This future research may also develop our understanding regarding the lack of association between polypharmacy and ischemic stroke, in AF.

## AUTHOR CONTRIBUTIONS

All authors contributed to the study idea. Martin Frisher led the study and Natasha Slater conducted all data analysis, and they take full responsibility for the integrity and accuracy of the analysis. All authors contributed to data interpretation. Natasha Slater drafted the manuscript with contributions from Martin Frisher and Simon White. Natasha Slater and Martin Frisher are the guarantors for this study.

## FUNDING INFORMATION

This research received no specific grant from any funding agency in the public, commercial, or not‐for‐profit sectors.

## CONFLICT OF INTEREST STATEMENT

All authors declare that there is nothing to disclose.

## COPYRIGHT/LICENSE FOR PUBLICATION

The corresponding author has the right to grant on behalf of all authors and does grant on behalf of all authors, *a worldwide license* to the Publishers and its licensees in perpetuity, in all forms, formats, and media (whether known now or created in the future), to (i) publish, reproduce, distribute, display, and store the Contribution, (ii) translate the Contribution into other languages, create adaptations, reprints, include within collections and create summaries, extracts and/or, abstracts of the Contribution, (iii) create any other derivative work(s) based on the Contribution, (iv) exploit all subsidiary rights in the Contribution, (v) the inclusion of electronic links from the Contribution to third party material where‐ever it may be located; and, and (vi) license any third party to do any or all of the above.

## Supporting information


Data S1:

## Data Availability

Data may be obtained from a third party and are not publicly available. The data were obtained from the Clinical Practice Research Datalink (CPRD). CPRD data governance does not allow us to distribute patient data to other parties. Researchers may apply for data access at http://www.CPRD.com/.
