# Supplementary material for: Polypharmacy in atrial fibrillation: A prospective analysis of mortality and ischemic stroke using the Clinical Practice Research Datalink
Source: J Arrhythm. 2023 Nov 27;40(1):47–56. doi: 10.1002/joa3.12961 (PMC10848617; doi:10.1002/joa3.12961)

## **Supplementary Material:**

## **Read code lists for atrial fibrillation, ischaemic stroke, and all prognostic factors.**

Table S1: Read code list for atrial fibrillation.

| **Pegasus Dictionary Code** | **Read Code** | **Description** |
| --- | --- | --- |
| 1268 | G573200 | Paroxysmal AF |
| 1664 | G573000 | Atrial fibrillation (AF) |
| 2212 | G573.00 | AF with flutter |
| 3757 | 3272.00 | ECG: Atrial fibrillation |
| 23437 | G573z00 | AF and flutter NOS (no other symptoms) |
| 35127 | G573300 | Non-rheumatic AF |
| 96076 | G573500 | Persistent AF |
| 96277 | G573400 | Permanent AF |

Table S2: Read code list for ischaemic stroke.

| **Pegasus Dictionary Code** | **Read Code** | **Description** |
| --- | --- | --- |
| 504 | G65..00 | Transient cerebral ischaemia |
| 569 | G64..12 | Infarction - cerebral |
| 1298 | G66..11 | Cerebrovascular accident (CVA) -not specified |
| 1433 | G65..12 | Transient ischaemic attack |
| 1469 | G66..00 | Stroke and CVA |
| 1895 | G65z.00 | Transient cerebral ischaemia NOS |
| 2417 | G65..13 | Vertebro-basilar insufficiency |
| 3149 | G64z.00 | Cerebral infarction NOS |
| 3979 | G672.00 | Hypertensive encephalopathy |
| 4152 | G631.12 | Thrombosis, carotid artery |
| 4240 | G631.00 | Carotid artery occlusion |
| 5184 | G670.11 | Precerebral atherosclerosis |
| 5185 | G64z111 | Lateral medullary syndrome |
| 5268 | G650.11 | Insufficiency - basilar artery |
| 5363 | G64..11 | CVA – cerebral artery occlusion |
| 5602 | G64z.12 | Cerebellar infarction |
| 6116 | G66..13 | CVA- cerebrovascular accident – not specified |
| 6155 | G64..13 | Stroke due to cerebral arterial occlusion |
| 6228 | G68x.00 | Sequelae of stroke – not specified as haemorrhagic or ischaemic |
| 6253 | G66..12 | Stroke – not specified |
| 6489 | G655.00 | Transient global amnesia |
| 7780 | G667.00 | Left-sided CVA |
| 8443 | G663.00 | Brain stem stroke syndrome |
| 8837 | G64..00 | Cerebral arterial occlusion |
| 9985 | G642200 | Left-sided cerebral infarction |
| 10062 | G6z..00 | Cerebrovascular disease NOS |
| 10504 | G64z300 | Right-sided cerebral infarction |
| 11171 | G670.00 | Cerebral atherosclerosis |
| 12555 | G671z00 | Generalised ischaemic cerebrovascular disease NOS |
| 12833 | G668.00 | Right-sided CVA |
| 13577 | G67..00 | Other cerebrovascular disease |
| 15019 | G641.00 | Cerebral embolism |
| 15252 | G64z.11 | Brainstem infarction NOS |
| 15788 | G65zz00 | Transient cerebral ischaemia NOS |
| 16507 | G65z100 | Intermittent cerebral ischaemia |
| 16517 | G640.00 | Cerebral thrombosis |
| 17322 | G664.00 | Cerebellar stroke |
| 18689 | G660.00 | Middle cerebral artery syndrome |
| 19260 | G662.00 | Posterior cerebral artery syndrome |
| 19280 | G661.00 | Anterior cerebral artery syndrome |
| 19348 | ZV12511 | [V]Personal history of stroke |
| 19354 | G65y.00 | Other transient cerebral ischaemia |
| 21118 | G651000 | Vertebro-basilar artery syndrome |
| 23465 | G652.00 | Subclavian steal syndrome |
| 23671 | G63y000 | Cerebral infarction due to thrombosis of the pre-cerebral arteries |
| 23942 | G650.00 | Basilar artery syndrome |
| 24385 | G671100 | Chronic cerebral ischaemia |
| 24446 | G63y100 | Cerebral infarction due to embolism in the pre-cerebral arteries |
| 25615 | G64z000 | Brainstem infarction |
| 27975 | G641000 | Cerebral infarction due to embolism in the cerebral arteries |
| 32447 | G630.00 | Basilar artery occlusion |
| 33377 | G651.00 | Vertebral artery syndrome |
| 33499 | G665.00 | Pure motor lacunar syndrome |
| 33543 | G6X..00 | Cerebrl infarctn due/unspcf occlusn or sten/cerebrl artrs |
| 34117 | G67y.00 | Other cerebrovascular disease OS |
| 34758 | G641.11 | Cerebral embolus |
| 36717 | G640000 | Cerebral infarction due to thrombosis of the cerebral arteries |
| 37493 | G67z.00 | Other cerebrovascular disease NOS |
| 39344 | G676000 | Cerebral infarction due to cerebral venous thrombosis |
| 39403 | G683.00 | Sequelae of cerebral infarction |
| 40053 | G671.00 | Generalised ischaemic cerebrovascular disease NOS |
| 40758 | G6W..00 | Cereb infarct due unsp occlus/stenos precerebr arteries |
| 40847 | G632.00 | Vertebral artery occlusion |
| 44765 | G653.00 | Carotid artery syndrome hemispheric |
| 45781 | G63..00 | Precerebral arterial occlusion |
| 47642 | G64z100 | Wallenberg syndrome |
| 50594 | G654.00 | Multiple and bilateral precerebral artery syndromes |
| 51311 | G6y..00 | Other specified cerebrovascular disease |
| 51326 | G63y.00 | Other precerebral artery occlusion |
| 51767 | G666.00 | Pure sensory lacunar syndrome |
| 53475 | Gyu6400 | Cerebral infarction (other) |
| 55247 | G65z000 | Impending cerebral ischaemia |
| 57495 | G63..11 | Infarction - precerebral |
| 70536 | G671000 | Acute cerebrovascular insufficiency NOS |
| 71585 | G63z.00 | Precerebral artery occlusion NOS |
| 73901 | Gyu6.00 | [X]Cerebrovascular diseases |
| 91627 | Gyu6300 | [X]Cerebrl infarctn due/unspcf occlusn or sten/cerebrl artrs |
| 94482 | Gyu6G00 | [X]Cereb infarct due unsp occlus/stenos precerebr arteries |
| 98642 | G633.00 | Multiple and bilateral precerebral arterial occlusion |

Table S3: Read code list for Body Mass Index (BMI)

| **Pegasus Dictionary Code** | **Read Code** | **Description** |
| --- | --- | --- |
| 9015 | 22K4.00 | BMI 25-29 (overweight) |
| 13278 | 22K5.00 | BMI 30+ (obese) |
| 22556 | 22K7.00 | BMI >40 (severely obese) |
| 24498 | 22K6.00 | BMI <20 |
| 28937 | 22K2.00 | BMI high |
| 28946 | 22K1.00 | BMI normal |
| 44291 | 22K8.00 | BMI 20-24 |

Note: Actual BMI values will be obtained from additional file in patient records via enttype 13 = weight and data 3 =BMI

Table S4: Read code list for hypertension

| **Pegasus Dictionary Code** | **Read Code** | **Description** |
| --- | --- | --- |
| 204 | G2...00 | Hypertensive disease |
| 351 | G20..11 | High blood pressure |
| 799 | G20..00 | Essential hypertension |
| 1894 | G201.00 | Benign essential hypertension |
| 3425 | 662O.00 | On treatment for hypertension |
| 3712 | G20z.11 | Hypertension NOS |
| 4372 | G202.00 | Systolic hypertension |
| 4668 | G22..00 | Hypertensive renal disease |
| 7057 | G2z..00 | Hypertensive disease NOS |
| 7329 | G24..00 | Secondary hypertension |
| 8732 | G2...11 | BP - hypertensive disease |
| 8857 | G21z011 | Cardiomegaly - hypertensive |
| 10818 | G20z.00 | Essential hypertension NOS |
| 13188 | 662G.00 | Hypertensive treatm.changed |
| 15106 | G22z.00 | Hypertensive renal disease NOS |
| 15377 | G200.00 | Malignant essential hypertension |
| 16059 | G24z.00 | Secondary hypertension NOS |
| 16173 | G21zz00 | Hypertensive heart disease NOS |
| 16292 | G21..00 | Hypertensive heart disease |
| 18765 | G2y..00 | Other specified hypertensive disease |
| 21826 | 662F.00 | Hypertension treatm. started |
| 21837 | G232.00 | Hypertensive heart&renal dis wth (congestive) heart failure |
| 25371 | G241000 | Secondary benign renovascular hypertension |
| 27511 | 6628 | Poor hypertension control |
| 28684 | G233.00 | Hypertensive heart and renal disease with renal failure |
| 29310 | G22z.11 | Renal hypertension |
| 31341 | G24z100 | Hypertension secondary to drug |
| 31387 | G24z000 | Secondary renovascular hypertension NOS |
| 31464 | G21z.00 | Hypertensive heart disease NOS |
| 31755 | G240.00 | Secondary malignant hypertension |
| 31816 | G672.11 | Hypertensive crisis |
| 32423 | G222.00 | Hypertensive renal disease with renal failure |
| 34744 | G244.00 | Hypertension secondary to endocrine disorders |
| 39649 | G220.00 | Malignant hypertensive renal disease |
| 42229 | G24zz00 | Secondary hypertension NOS |
| 43935 | G221.00 | Benign hypertensive renal disease |
| 50157 | G210.00 | Malignant hypertensive heart disease |
| 51635 | G241z00 | Secondary benign hypertension NOS |
| 52127 | G211100 | Benign hypertensive heart disease with CCF |
| 52427 | G211.00 | Benign hypertensive heart disease |
| 57288 | G241.00 | Secondary benign hypertension |
| 57987 | G234.00 | Hyperten heart&renal dis+both(congestv)heart and renal fail |
| 59383 | G240000 | Secondary malignant renovascular hypertension |
| 61166 | G21z000 | Hypertensive heart disease NOS without CCF |
| 61660 | G211000 | Benign hypertensive heart disease without CCF |
| 62718 | G21z100 | Hypertensive heart disease NOS with CCF |
| 63000 | G231.00 | Benign hypertensive heart and renal disease |
| 63466 | G23..00 | Hypertensive heart and renal disease |
| 67232 | G230.00 | Malignant hypertensive heart and renal disease |
| 68659 | G23z.00 | Hypertensive heart and renal disease NOS |
| 69753 | Gyu2.00 | [X]Hypertensive diseases |
| 72668 | G210100 | Malignant hypertensive heart disease with CCF |
| 73293 | G240z00 | Secondary malignant hypertension NOS |
| 95334 | G210000 | Malignant hypertensive heart disease without CCF |
| 97533 | Gyu2100 | [X]Hypertension secondary to other renal disorders |
| 102458 | Gyu2000 | [X]Other secondary hypertension |

Table S5: Read code list for diabetes mellitus

| **Pegasus Dictionary Code** | **Read Code** | **Description** |
| --- | --- | --- |
| 506 | C100112 | Non-insulin dependent diabetes mellitus |
| 711 | C10..00 | Diabetes mellitus |
| 758 | C10F.00 | Type 2 diabetes mellitus |
| 1038 | C100011 | Insulin dependent diabetes mellitus |
| 1045 | C135.00 | Diabetes insipidus |
| 1407 | C10FJ00 | Insulin treated Type 2 diabetes mellitus |
| 1549 | C10E.00 | Type 1 diabetes mellitus |
| 1647 | C108.00 | Insulin dependent diabetes mellitus |
| 1682 | C101.00 | Diabetes mellitus with ketoacidosis |
| 2378 | 66AJ.00 | Diabetic - poor control |
| 2471 | K01x100 | Nephrotic syndrome in diabetes mellitus |
| 2478 | 66AJ100 | Brittle diabetes |
| 4513 | C109.00 | Non-insulin dependent diabetes mellitus |
| 5884 | C109.11 | NIDDM - Non-insulin dependent diabetes mellitus |
| 6430 | 9NM0.00 | Attending diabetes clinic |
| 6509 | C108700 | Insulin dependent diabetes mellitus with retinopathy |
| 6791 | C108800 | Insulin dependent diabetes mellitus - poor control |
| 6813 | 1434 | H/O: diabetes mellitus |
| 7045 | 14F4.00 | H/O: Admission in last year for diabetes foot problem |
| 7563 | 66A3.00 | Diabetic on diet only |
| 7795 | C106.12 | Diabetes mellitus with neuropathy |
| 8306 | 8H7f.00 | Referral to diabetes nurse |
| 8403 | C109700 | Diabetes mellitus with neuropathy |
| 8836 | 66AR.00 | Diabetes management plan given |
| 8842 | 66A5.00 | Diabetic on insulin |
| 9013 | 66AJ.11 | Unstable diabetes |
| 9897 | 9OL..00 | Diabetes monitoring admin. |
| 10098 | C10yy00 | Other specified diabetes mellitus with other spec comps |
| 10418 | C10ED00 | Type 1 diabetes mellitus with nephropathy |
| 10642 | ZC2C800 | Dietary advice for diabetes mellitus |
| 10692 | C10EM00 | Type 1 diabetes mellitus with ketoacidosis |
| 11359 | L180.00 | Diabetes mellitus during pregnancy/childbirth/puerperium |
| 11471 | 8B3l.00 | Diabetes medication review |
| 11551 | C10B.00 | Diabetes mellitus induced by steroids |
| 11599 | 7276 | Pan retinal photocoagulation for diabetes |
| 11848 | C314.11 | Renal diabetes |
| 11930 | 9NN9.00 | Under care of diabetes specialist nurse |
| 11977 | ZL62500 | Referral to diabetes nurse |
| 12030 | 9OL6.00 | Diabetes monitoring 3rd letter |
| 12213 | 8BL2.00 | Patient on maximal tolerated therapy for diabetes |
| 12307 | 66AU.00 | Diabetes care by hospital only |
| 12455 | C10E.11 | Type I diabetes mellitus |
| 12506 | 66AP.00 | Diabetes: practice programme |
| 12640 | C10FC00 | Type 2 diabetes mellitus with nephropathy |
| 12675 | 66AQ.00 | Diabetes: shared care programme |
| 12682 | 679R.00 | Patient offered diabetes structured education programme |
| 12703 | 3881 | Education score - diabetes |
| 12736 | C10F500 | Type 2 diabetes mellitus with gangrene |
| 13057 | 679L.00 | Health education - diabetes |
| 13069 | 66A8.00 | Has seen dietician - diabetes |
| 13191 | 9OL..11 | Diabetes clinic administration |
| 13192 | 9OLA.00 | Diabetes monitor. check done |
| 13194 | 9OL4.00 | Diabetes monitoring 1st letter |
| 13195 | 9OL5.00 | Diabetes monitoring 2nd letter |
| 13197 | 9OL1.00 | Attends diabetes monitoring |
| 13279 | C104y00 | Other specified diabetes mellitus with renal complications |
| 13071 | 66AI.00 | Diabetic - good control |
| 14889 | C100111 | Maturity onset diabetes |
| 14803 | C100100 | Diabetes mellitus, adult onset, no mention of complication |
| 15690 | C103.00 | Diabetes mellitus with ketoacidotic coma |
| 16230 | C106.00 | Diabetes mellitus with neurological manifestation |
| 16491 | C106.13 | Diabetes mellitus with polyneuropathy |
| 16502 | C104.00 | Diabetes mellitus with renal manifestation |
| 16881 | ZV65312 | [V]Dietary counselling in diabetes mellitus |
| 17067 | F171100 | Autonomic neuropathy due to diabetes |
| 17262 | C109600 | Non-insulin-dependent diabetes mellitus with retinopathy |
| 17545 | C108F11 | Type I diabetes mellitus with diabetic cataract |
| 17858 | C108.12 | Type 1 diabetes mellitus |
| 17859 | C109.12 | Type 2 diabetes mellitus |
| 17869 | 66AL.00 | Diabetic-uncooperative patient |
| 18056 | 2G5C.00 | Foot abnormality - diabetes related |
| 18143 | C109G11 | Type II diabetes mellitus with arthropathy |
| 18209 | C109012 | Type 2 diabetes mellitus with renal complications |
| 18219 | C109.13 | Type II diabetes mellitus |
| 18230 | C108J12 | Type 1 diabetes mellitus with neuropathic arthropathy |
| 18264 | C109J12 | Insulin treated Type II diabetes mellitus |
| 18278 | C109J00 | Insulin treated Type 2 diabetes mellitus |
| 18387 | B906800 | Type 1 diabetes mellitus with retinopathy |
| 18390 | C10FM00 | Type 2 diabetes mellitus with persistent microalbuminuria |
| 18425 | C10FB00 | Type 2 diabetes mellitus with polyneuropathy |
| 18496 | C10F600 | Type 2 diabetes mellitus with retinopathy |
| 18505 | C108.11 | IDDM-Insulin dependent diabetes mellitus |
| 18642 | C10EH00 | Type 1 diabetes mellitus with arthropathy |
| 18683 | C10E500 | Type 1 diabetes mellitus with ulcer |
| 18766 | 212H.00 | Diabetes resolved |
| 18777 | C10F000 | Type 2 diabetes mellitus with renal complications |
| 20900 | 9OLA.11 | Diabetes monitored |
| 21482 | C102.00 | Diabetes mellitus with hyperosmolar coma |
| 21983 | C108012 | Type 1 diabetes mellitus with renal complications |
| 22487 | C10N.00 | Secondary diabetes mellitus |
| 22023 | 66AJz00 | Diabetic - poor control NOS |
| 22573 | C106z00 | Diabetes mellitus NOS with neurological manifestation |
| 22871 | C10EP00 | Type 1 diabetes mellitus with exudative maculopathy |
| 22884 | C10F.11 | Type II diabetes mellitus |
| 22967 | 2BBF.00 | Retinal abnormality - diabetes related |
| 23479 | C350011 | Bronzed diabetes |
| 24423 | C108.13 | Type I diabetes mellitus |
| 24458 | C109711 | Type II diabetes mellitus - poor control |
| 24490 | C100000 | Diabetes mellitus, juvenile type, no mention of complication |
| 24693 | C109G00 | Non-insulin dependent diabetes mellitus with arthropathy |
| 24694 | C108B00 | Insulin dependent diabetes mellitus with mononeuropathy |
| 24836 | C109C12 | Type 2 diabetes mellitus with nephropathy |
| 25041 | ZC2CA00 | Dietary advice for type II diabetes |
| 25591 | C10FQ00 | Type 2 diabetes mellitus with exudative maculopathy |
| 25627 | C10F700 | Type 2 diabetes mellitus - poor control |
| 26054 | C10FL00 | Type 2 diabetes mellitus with persistent proteinuria |
| 26108 | C10B000 | Steroid induced diabetes mellitus without complication |
| 26605 | 9OLB.00 | Attended diabetes structured education programme |
| 26855 | C108400 | Unstable insulin dependent diabetes mellitus |
| 27921 | 2G51000 | Foot abnormality - diabetes related |
| 28622 | 2126300 | Diabetes resolved |
| 28856 | 8CP2.00 | Transition of diabetes care options discussed |
| 29979 | C109900 | Non-insulin-dependent diabetes mellitus without complication |
| 30294 | C10EL00 | Type 1 diabetes mellitus with persistent microalbuminuria |
| 30323 | C10EK00 | Type 1 diabetes mellitus with persistent proteinuria |
| 30970 | Q44B.00 | Syndrome of infant of mother with gestational diabetes |
| 31141 | 9OL8.00 | Diabetes monitor.phone invite |
| 31240 | 9OL7.00 | Diabetes monitor.verbal invite |
| 31241 | 9OLZ.00 | Diabetes monitoring admin.NOS |
| 31310 | C108900 | Insulin dependent diabetes maturity onset |
| 31790 | F372.00 | Polyneuropathy in diabetes |
| 32193 | C11y000 | Steroid induced diabetes |
| 32359 | ZRbH.00 | Perceived control of insulin-dependent diabetes |
| 32403 | C107.11 | Diabetes mellitus with gangrene |
| 32556 | C107.12 | Diabetes with gangrene |
| 32619 | 66Af.00 | Patient diabetes education review |
| 32627 | C10FN00 | Type 2 diabetes mellitus with ketoacidosis |
| 32739 | 9N0n.00 | Seen in community diabetes specialist clinic |
| 33254 | C105.00 | Diabetes mellitus with ophthalmic manifestation |
| 33343 | C10y.00 | Diabetes mellitus with other specified manifestation |
| 33807 | C107200 | Diabetes mellitus, adult with gangrene |
| 33969 | C10A100 | Malnutrition-related diabetes mellitus with ketoacidosis |
| 34268 | C10F200 | Type 2 diabetes mellitus with neurological complications |
| 34283 | C105z00 | Diabetes mellitus NOS with ophthalmic manifestation |
| 34450 | C10FK00 | Hyperosmolar non-ketotic state in type 2 diabetes mellitus |
| 34912 | C109400 | Non-insulin dependent diabetes mellitus with ulcer |
| 35105 | C104100 | Diabetes mellitus, adult onset, with renal manifestation |
| 35107 | C104z00 | Diabetes mellitus with nephropathy NOS |
| 35288 | C10E800 | Type 1 diabetes mellitus - poor control |
| 35385 | C10FH00 | Type 2 diabetes mellitus with neuropathic arthropathy |
| 35399 | C107.00 | Diabetes mellitus with peripheral circulatory disorder |
| 36633 | C109K00 | Hyperosmolar non-ketotic state in type 2 diabetes mellitus |
| 36695 | C10D.00 | Diabetes mellitus autosomal dominant type 2 |
| 37648 | C109J11 | Insulin treated non-insulin dependent diabetes mellitus |
| 37806 | C10FF00 | Type 2 diabetes mellitus with peripheral angiopathy |
| 38078 | 66A9.00 | Understands diet - diabetes |
| 38161 | C108711 | Type I diabetes mellitus with retinopathy |
| 38617 | C101y00 | Other specified diabetes mellitus with ketoacidosis |
| 38986 | C100.00 | Diabetes mellitus with no mention of complication |
| 39070 | C10EE00 | Type 1 diabetes mellitus with hypoglycaemic coma |
| 39317 | C106100 | Diabetes mellitus, adult onset, + neurological manifestation |
| 39809 | C108J00 | Insulin dependent diab mell with neuropathic arthropathy |
| 40023 | C102000 | Diabetes mellitus, juvenile type, with hyperosmolar coma |
| 40401 | C109500 | Non-insulin dependent diabetes mellitus with gangrene |
| 40682 | C10E900 | Type 1 diabetes mellitus maturity onset |
| 40837 | C10EN00 | Type 1 diabetes mellitus with ketoacidotic coma |
| 41049 | C108712 | Type 1 diabetes mellitus with retinopathy |
| 41389 | C105100 | Diabetes mellitus, adult onset, + ophthalmic manifestation |
| 41686 | Cyu2000 | [X]Other specified diabetes mellitus |
| 41716 | C108C00 | Insulin dependent diabetes mellitus with polyneuropathy |
| 42505 | C101z00 | Diabetes mellitus NOS with ketoacidosis |
| 42567 | C103000 | Diabetes mellitus, juvenile type, with ketoacidotic coma |
| 42729 | C108E11 | Type I diabetes mellitus with hypoglycaemic coma |
| 42762 | C109612 | Type 2 diabetes mellitus with retinopathy |
| 42831 | C10E200 | Type 1 diabetes mellitus with neurological complications |
| 43139 | C102100 | Diabetes mellitus, adult onset, with hyperosmolar coma |
| 43453 | C10C.00 | Diabetes mellitus autosomal dominant |
| 43785 | C109D00 | Non-insulin dependent diabetes mellitus with hypoglyca coma |
| 43857 | C10M.00 | Lipoatrophic diabetes mellitus |
| 43921 | C10E400 | Unstable type 1 diabetes mellitus |
| 44260 | C108F00 | Insulin dependent diabetes mellitus with diabetic cataract |
| 44312 | 9M10.00 | Informed dissent for diabetes national audit |
| 44440 | C108E00 | Insulin dependent diabetes mellitus with hypoglycaemic coma |
| 44443 | C108500 | Insulin dependent diabetes mellitus with ulcer |
| 44779 | C109E12 | Type 2 diabetes mellitus with diabetic cataract |
| 44982 | C10FE00 | Type 2 diabetes mellitus with diabetic cataract |
| 45467 | C109B00 | Non-insulin dependent diabetes mellitus with polyneuropathy |
| 45491 | C10z.00 | Diabetes mellitus with unspecified complication |
| 45913 | C109712 | Type 2 diabetes mellitus - poor control |
| 45914 | C108812 | Type 1 diabetes mellitus - poor control |
| 45919 | C109212 | Type 2 diabetes mellitus with neurological complications |
| 46150 | C109512 | Type 2 diabetes mellitus with gangrene |
| 46290 | C108y00 | Other specified diabetes mellitus with multiple comps |
| 46301 | C10EC00 | Type 1 diabetes mellitus with polyneuropathy |
| 46577 | 66AX.00 | Diabetes: shared care in pregnancy - diabetol and obstet |
| 46624 | C10C.11 | Maturity onset diabetes in youth |
| 46850 | C108811 | Type I diabetes mellitus - poor control |
| 46917 | C10FD00 | Type 2 diabetes mellitus with hypoglycaemic coma |
| 46963 | C108000 | Insulin-dependent diabetes mellitus with renal complications |
| 47011 | 8Hj0.00 | Referral to diabetes structured education programme |
| 47032 | 8CS0.00 | Diabetes care plan agreed |
| 47058 | 8Hg4.00 | Discharged from care of diabetes specialist nurse |
| 47315 | C10F711 | Type II diabetes mellitus - poor control |
| 47321 | C10F100 | Type 2 diabetes mellitus with ophthalmic complications |
| 47377 | C105y00 | Other specified diabetes mellitus with ophthalmic complicatn |
| 47409 | C109B11 | Type II diabetes mellitus with polyneuropathy |
| 47582 | C10E000 | Type 1 diabetes mellitus with renal complications |
| 47649 | C10E100 | Type 1 diabetes mellitus with ophthalmic complications |
| 47650 | C10E300 | Type 1 diabetes mellitus with multiple complications |
| 47816 | C109H11 | Type II diabetes mellitus with neuropathic arthropathy |
| 47954 | C10F900 | Type 2 diabetes mellitus without complication |
| 48192 | C109E11 | Type II diabetes mellitus with diabetic cataract |
| 49074 | C10F400 | Type 2 diabetes mellitus with ulcer |
| 49146 | C108211 | Type I diabetes mellitus with neurological complications |
| 49276 | C108100 | Insulin-dependent diabetes mellitus with ophthalmic comps |
| 49554 | C10EF00 | Type 1 diabetes mellitus with diabetic cataract |
| 49869 | C109G12 | Type 2 diabetes mellitus with arthropathy |
| 49949 | C10E411 | Unstable type I diabetes mellitus |
| 50225 | C109011 | Type II diabetes mellitus with renal complications |
| 50429 | C109100 | Non-insulin-dependent diabetes mellitus with ophthalm comps |
| 50609 | L180600 | Pre-existing diabetes mellitus, non-insulin-dependent |
| 50813 | C109A11 | Type II diabetes mellitus with mononeuropathy |
| 50937 | 8HTe.00 | Referral to diabetes preconception counselling clinic |
| 50960 | L180500 | Pre-existing diabetes mellitus, insulin-dependent |
| 50972 | C100z00 | Diabetes mellitus NOS with no mention of complication |
| 51261 | C10E.12 | Insulin dependent diabetes mellitus |
| 51697 | C10G.00 | Secondary pancreatic diabetes mellitus |
| 51756 | C10FP00 | Type 2 diabetes mellitus with ketoacidotic coma |
| 51957 | C108511 | Type I diabetes mellitus with ulcer |
| 52104 | C108300 | Insulin dependent diabetes mellitus with multiple complication |
| 52212 | Cyu2.00 | [X]Diabetes mellitus |
| 52236 | C10A.00 | Malnutrition-related diabetes mellitus |
| 52283 | C108200 | Insulin-dependent diabetes mellitus with neurological comps |
| 52303 | C109000 | Non-insulin-dependent diabetes mellitus with renal comps |
| 53200 | C101000 | Diabetes mellitus, juvenile type, with ketoacidosis |
| 54008 | C10EJ00 | Type 1 diabetes mellitus with neuropathic arthropathy |
| 54419 | 918T.00 | Diabetes key contact |
| 54856 | C101100 | Diabetes mellitus, adult onset, with ketoacidosis |
| 54899 | C109F11 | Type II diabetes mellitus with peripheral angiopathy |
| 55075 | C109411 | Type II diabetes mellitus with ulcer |
| 55329 | C10EQ00 | Type 1 diabetes mellitus with gastroparesis |
| 55431 | L180X00 | Pre-existing diabetes mellitus, unspecified |
| 55842 | C109200 | Non-insulin-dependent diabetes mellitus with neuro comps |
| 56268 | C109D11 | Type II diabetes mellitus with hypoglycaemic coma |
| 56448 | C108A00 | Insulin-dependent diabetes without complication |
| 57621 | C108D00 | Insulin dependent diabetes mellitus with nephropathy |
| 58604 | C109611 | Type II diabetes mellitus with retinopathy |
| 59253 | C10FG00 | Type 2 diabetes mellitus with arthropathy |
| 59288 | C103y00 | Other specified diabetes mellitus with coma |
| 59365 | C109C00 | Non-insulin dependent diabetes mellitus with nephropathy |
| 59725 | C109111 | Type II diabetes mellitus with ophthalmic complications |
| 59991 | C10D.11 | Maturity onset diabetes in youth type 2 |
| 60107 | C108411 | Unstable type I diabetes mellitus |
| 60499 | C108600 | Insulin dependent diabetes mellitus with gangrene |
| 60699 | C109F12 | Type 2 diabetes mellitus with peripheral angiopathy |
| 60796 | C10FL11 | Type II diabetes mellitus with persistent proteinuria |
| 61071 | C109D12 | Type 2 diabetes mellitus with hypoglycaemic coma |
| 61122 | C10H.00 | Diabetes mellitus induced by non-steroid drugs |
| 61344 | C108011 | Type I diabetes mellitus with renal complications |
| 61461 | 9M00.00 | Informed consent for diabetes national audit |
| 61523 | C106y00 | Other specified diabetes mellitus with neurological comps |
| 61829 | C108212 | Type 1 diabetes mellitus with neurological complications |
| 62107 | C109511 | Type II diabetes mellitus with gangrene |
| 62146 | C109300 | Non-insulin-dependent diabetes mellitus with multiple comps |
| 62209 | C10EM11 | Type I diabetes mellitus with ketoacidosis |
| 62352 | C108H11 | Type I diabetes mellitus with arthropathy |
| 62674 | C10FA00 | Type 2 diabetes mellitus with mononeuropathy |
| 63017 | C108911 | Type I diabetes mellitus maturity onset |
| 63357 | C107100 | Diabetes mellitus, adult, + peripheral circulatory disorder |
| 63371 | C10y100 | Diabetes mellitus, adult, + other specified manifestation |
| 63412 | 8CR2.00 | Diabetes clinical management plan |
| 63690 | C10FR00 | Type 2 diabetes mellitus with gastroparesis |
| 63762 | C10z100 | Diabetes mellitus, adult onset, + unspecified complication |
| 64283 | C10zy00 | Other specified diabetes mellitus with unspecified comps |
| 64357 | C10zz00 | Diabetes mellitus NOS with unspecified complication |
| 64384 | L180z00 | Diabetes mellitus in pregnancy/childbirth/puerperium NOS |
| 64449 | C108z00 | Unspecified diabetes mellitus with multiple complications |
| 64571 | C109C11 | Type II diabetes mellitus with nephropathy |
| 65025 | C107z00 | Diabetes mellitus NOS with peripheral circulatory disorder |
| 65062 | C103z00 | Diabetes mellitus NOS with ketoacidotic coma |
| 65267 | C10F300 | Type 2 diabetes mellitus with multiple complications |
| 65616 | C108H00 | Insulin dependent diabetes mellitus with arthropathy |
| 65704 | C109412 | Type 2 diabetes mellitus with ulcer |
| 66675 | C10A000 | Malnutrition-related diabetes mellitus with coma |
| 66872 | C108D11 | Type I diabetes mellitus with nephropathy |
| 66965 | C109H12 | Type 2 diabetes mellitus with neuropathic arthropathy |
| 67635 | L180000 | Diabetes mellitus - unspec whether in pregnancy/puerperium |
| 67853 | C106000 | Diabetes mellitus, juvenile, + neurological manifestation |
| 67905 | C109211 | Type II diabetes mellitus with neurological complications |
| 68105 | C10EB00 | Type 1 diabetes mellitus with mononeuropathy |
| 68390 | C108512 | Type 1 diabetes mellitus with ulcer |
| 68546 | ZRB4.00 | Diabetes clinic satisfaction questionnaire |
| 68792 | C10z000 | Diabetes mellitus, juvenile type, + unspecified complication |
| 68818 | ZRB5.11 | DTSQ - Diabetes treatment satisfaction questionnaire |
| 68843 | C103100 | Diabetes mellitus, adult onset, with ketoacidotic coma |
| 69043 | ZC2C900 | Dietary advice for type I diabetes |
| 69278 | C109E00 | Non-insulin depend diabetes mellitus with diabetic cataract |
| 69676 | C10EA00 | Type 1 diabetes mellitus without complication |
| 69748 | C105000 | Diabetes mellitus, juvenile type, + ophthalmic manifestation |
| 69993 | C10E600 | Type 1 diabetes mellitus with gangrene |
| 70316 | C109112 | Type 2 diabetes mellitus with ophthalmic complications |
| 70448 | C107000 | Diabetes mellitus, juvenile +peripheral circulatory disorder |
| 70766 | C108E12 | Type 1 diabetes mellitus with hypoglycaemic coma |
| 70821 | C10yz00 | Diabetes mellitus NOS with other specified manifestation |
| 72320 | C109A00 | Non-insulin dependent diabetes mellitus with mononeuropathy |
| 72345 | C102z00 | Diabetes mellitus NOS with hyperosmolar coma |
| 91646 | C10F411 | Type II diabetes mellitus with ulcer |
| 93727 | C10FE11 | Type II diabetes mellitus with diabetic cataract |
| 93875 | C10E712 | Insulin dependent diabetes mellitus with retinopathy |
| 93922 | C104000 | Diabetes mellitus, juvenile type, with renal manifestation |
| 95343 | C10E711 | Type I diabetes mellitus with retinopathy |
| 95994 | 66Aq.00 | Diabetic foot screen |
| 96010 | 66Ap.00 | Insulin treatment initiated |
| 97474 | C108412 | Unstable type 1 diabetes mellitus |
| 97849 | C10E912 | Insulin dependent diabetes maturity onset |
| 97894 | C10EP11 | Type I diabetes mellitus with exudative maculopathy |
| 98071 | C10E112 | Insulin-dependent diabetes mellitus with ophthalmic comps |
| 98704 | C10E512 | Insulin dependent diabetes mellitus with ulcer |
| 98954 | 3883 | Diabetes treatment satisfaction questionnaire |
| 99231 | C108B11 | Type I diabetes mellitus with mononeuropathy |
| 99311 | C10E111 | Type I diabetes mellitus with ophthalmic complications |
| 99628 | Kyu0300 | [X]Glomerular disorders in diabetes mellitus |
| 100292 | Cyu2300 | [X]Unspecified diabetes mellitus with renal complications |
| 100347 | C10A500 | Malnutritn-relat diabetes melitus wth periph circul complctn |
| 100791 | 66Ar.00 | Insulin treatment stopped |
| 100964 | C10F111 | Type II diabetes mellitus with ophthalmic complications |
| 102201 | C10FC11 | Type II diabetes mellitus with nephropathy |
| 102434 | 66Au.00 | Diabetic erectile dysfunction review |
| 102740 | C108112 | Type 1 diabetes mellitus with ophthalmic complications |
| 103798 | 9b92000 | Diabetic medicine |
| 106528 | C10FN11 | Type II diabetes mellitus with ketoacidosis |
| 108007 | C108311 | Type I diabetes mellitus with multiple complications |

Table S6: Read code list for heart failure

| **Pegasus Dictionary Code** | **Read Code** | **Description** |
| --- | --- | --- |
| 398 | G580.00 | Congestive heart failure |
| 884 | G581.00 | Left ventricular failure |
| 1223 | G58..11 | Cardiac failure |
| 2062 | G58..00 | Heart failure |
| 2906 | G580.11 | Congestive cardiac failure |
| 4024 | G58z.00 | Heart failure NOS |
| 5255 | G581000 | Acute left ventricular failure |
| 5942 | G581.13 | Impaired left ventricular function |
| 10079 | G580.12 | Right heart failure |
| 10154 | G580.13 | Right ventricular failure |
| 11424 | G580300 | Compensated cardiac failure |
| 13189 | 662g.00 | New York Heart Association classification - class II |
| 15058 | 14A6.00 | H/O: heart failure |
| 17278 | G58z.12 | Cardiac failure NOS |
| 18853 | 662f.00 | New York Heart Association classification - class I |
| 23707 | G580000 | Acute congestive heart failure |
| 27884 | G580200 | Decompensated cardiac failure |
| 27964 | G582.00 | Acute heart failure |
| 32671 | G580100 | Chronic congestive heart failure |

Table S7: Read code list for ischaemic heart disease and other cardiac ischaemic conditions.

| **Pegasus Dictionary Code** | **Read Code** | **Description** |
| --- | --- | --- |
| 240 | G3...00 | Ischaemic heart disease |
| 509 | G5y3.00 | Cardiomegaly |
| 562 | G5y3411 | Left ventricular hypertrophy |
| 1318 | G700.00 | Aortic atherosclerosis |
| 1344 | G340.12 | Coronary artery disease |
| 1414 | G33z300 | Angina on effort |
| 1430 | G33..00 | Angina pectoris |
| 1431 | G311.13 | Unstable angina |
| 1655 | G340.11 | Triple vessel disease of the heart |
| 1676 | G3z..00 | Ischaemic heart disease NOS |
| 1735 | G71..00 | Aortic aneurysm |
| 1792 | G3...13 | IHD - Ischaemic heart disease |
| 1876 | G714.00 | Abdominal aortic aneurysm without mention of rupture |
| 2155 | G341000 | Ventricular cardiac aneurysm |
| 2491 | G30..12 | Coronary thrombosis |
| 3729 | G5y3100 | Ventricular dilatation |
| 3999 | G340000 | Single coronary vessel disease |
| 2724 | G5y3400 | Ventricular hypertrophy |
| 4656 | G311.11 | Crescendo angina |
| 5254 | G340100 | Double coronary vessel disease |
| 5413 | G340.00 | Coronary atherosclerosis |
| 6331 | G341.00 | Aneurysm of heart |
| 6336 | 14A5.00 | H/O: angina pectoris |
| 6872 | G71z.00 | Aortic aneurysm NOS |
| 7320 | G343.00 | Ischaemic cardiomyopathy |
| 8568 | G37..00 | Cardiac syndrome X |
| 9276 | G31y000 | Acute coronary insufficiency |
| 9413 | G31y.00 | Other acute and subacute ischaemic heart disease |
| 11048 | G331.11 | Variant angina pectoris |
| 13571 | G30..16 | Thrombosis - coronary |
| 13578 | G5y3.11 | Dilatation - cardiac |
| 14904 | G5y3z00 | Cardiomegaly NOS |
| 15304 | G715.00 | Ruptured aortic aneurysm NOS |
| 15754 | G34z.00 | Other chronic ischaemic heart disease NOS |
| 16034 | G716.00 | Aortic aneurysm without mention of rupture NOS |
| 16521 | G710.00 | Dissecting aortic aneurysm |
| 16993 | 14AE.00 | H/O: aortic aneurysm |
| 18125 | G330000 | Nocturnal angina |
| 19655 | G311.14 | Angina at rest |
| 20416 | G3...12 | Atherosclerotic heart disease |
| 22383 | G3y..00 | Other specified ischaemic heart disease |
| 23078 | G34y100 | Chronic myocardial ischaemia |
| 24540 | G34y000 | Chronic coronary insufficiency |
| 24783 | G3...11 | Arteriosclerotic heart disease |
| 25842 | G33z.00 | Angina pectoris NOS |
| 27484 | G341.11 | Cardiac aneurysm |
| 27951 | G31..00 | Other acute and subacute ischaemic heart disease |
| 27977 | G31yz00 | Other acute and subacute ischaemic heart disease NOS |
| 28004 | G74..13 | Arterial embolic and thrombotic occlusion |
| 28062 | G743.00 | Embolism and thrombosis of other and unspec parts aorta |
| 28138 | G34..00 | Other chronic ischaemic heart disease |
| 28554 | G33zz00 | Angina pectoris NOS |
| 31900 | G740.11 | Aortic bifurcation syndrome |
| 34633 | G34y.00 | Other specified chronic ischaemic heart disease |
| 35713 | G34yz00 | Other specified chronic ischaemic heart disease NOS |
| 36609 | G342.00 | Atherosclerotic cardiovascular disease |
| 39449 | G312.00 | Coronary thrombosis not resulting in myocardial infarction |
| 39546 | Gyu3000 | [X]Other forms of angina pectoris |
| 41677 | G341z00 | Aneurysm of heart NOS |
| 42014 | G5y3200 | Cardiac dilatation NOS |
| 47637 | Gyu3300 | [X]Other forms of chronic ischaemic heart disease |
| 52517 | Gyu3.00 | [X]Ischaemic heart diseases |
| 56621 | G5y2.00 | Cardiovascular arteriosclerosis unspecified |
| 57062 | 14AJ.00 | H/O: Angina in last year |
| 61124 | G5y3500 | Cardiac hypertrophy NOS |
| 68401 | Gyu3200 | [X]Other forms of acute ischaemic heart disease |
| 70260 | G717.00 | Aortic aneurysm - syphilitic |
| 102719 | Gyu7200 | [X]Aortic aneurysm of unspecified site, nonruptured |
| 102725 | Gyu7100 | [X]Aortic aneurysm of unspecified site, ruptured |

Table S8: Read code list for myocardial infarction

| **Pegasus Dictionary Code** | **Read Code** | **Description** |
| --- | --- | --- |
| 241 | G30..00 | Acute myocardial infarction |
| 1204 | G30..14 | Heart attack |
| 1677 | G30..15 | MI - acute myocardial infarction |
| 1678 | G308.00 | Inferior myocardial infarction NOS |
| 2099 | G575.00 | Cardiac arrest |
| 3704 | G307.00 | Acute subendocardial infarction |
| 5387 | G301.00 | Other specified anterior myocardial infarction |
| 7783 | 323..00 | ECG: myocardial infarction |
| 8935 | G302.00 | Acute inferolateral infarction |
|  |  |  |
| 12139 | G300.00 | Acute anterolateral infarction |
|  |  |  |
| 13566 | G30..11 | Attack – heart |
| 14658 | G30z.00 | Acute myocardial infarction NOS |
| 14897 | G301z00 | Anterior myocardial infarction NOS |
| 14898 | G305.00 | Lateral myocardial infarction NOS |
| 16408 | G32..11 | Healed myocardial infarction |
| 17464 | G32..12 | Personal history of myocardial infarction |
| 17689 | G30..17 | Silent myocardial infarction |
| 17872 | G301100 | Acute anteroseptal infarction |
| 18842 | G35..00 | Subsequent myocardial infarction |
| 23708 | G361.00 | Atrial septal defect/curr comp folow acut myocardal infarct |
| 23892 | G304.00 | Posterior myocardial infarction NOS |
| 24126 | G360.00 | Haemopericardium/current comp folow acut myocard infarct |
| 26972 | 3234 | ECG:posterior/inferior infarct |
| 26975 | 3233 | ECG: antero-septal infarct. |
| 28736 | G30y000 | Acute atrial infarction |
| 29421 | G344.00 | Silent myocardial ischaemia |
| 29553 | G366.00 | Thrombosis atrium,auric append&vent/curr comp foll acute MI |
| 29643 | G303.00 | Acute inferoposterior infarction |
| 29758 | G30X.00 | Acute transmural myocardial infarction of unspecif site |
| 30421 | G30..13 | Cardiac rupture following myocardial infarction (MI) |
| 33402 | G575.12 | Asystole |
| 33899 | G575000 | Cardiac arrest with successful resuscitation |
| 34803 | G30y.00 | Other acute myocardial infarction |
| 35119 | G501.00 | Post infarction pericarditis |
| 35674 | 14A3.00 | H/O: myocardial infarct <60 |
| 36423 | G36..00 | Certain current complication follow acute myocardial infarct |
| 36523 | G311.00 | Preinfarction syndrome |
| 37657 | G362.00 | Ventric septal defect/curr comp fol acut myocardal infarctn |
| 38609 | G351.00 | Subsequent myocardial infarction of inferior wall |
| 39655 | G311.12 | Impending infarction |
| 3990 | 323 | ECG: old myocardial infarction |
| 40399 | 14A4.00 | H/O: myocardial infarct >60 |
| 40429 | G301000 | Acute anteroapical infarction |
| 41221 | G30y200 | Acute septal infarction |
| 45809 | G350.00 | Subsequent myocardial infarction of anterior wall |
| 46017 | G30yz00 | Other acute myocardial infarction NOS |
| 46166 | G35X.00 | Subsequent myocardial infarction of unspecified site |
| 50372 | 14AH.00 | H/O: Myocardial infarction in last year |
| 52705 | 3236 | ECG: lateral infarction |
| 54251 | G311z00 | Preinfarction syndrome NOS |
| 55401 | 3235 | ECG: subendocardial infarct |
| 59032 | 323Z.00 | ECG: myocardial infarct NOS |
| 59189 | G363.00 | Ruptur cardiac wall w'out haemopericard/cur comp fol ac MI |
| 59940 | G364.00 | Ruptur chordae tendinae/curr comp fol acute myocard infarct |
| 63467 | G306.00 | True posterior myocardial infarction |
| 68357 | G31y100 | Microinfarction of heart |
| 69474 | G365.00 | Rupture papillary muscle/curr comp fol acute myocard infarct |
| 72562 | G353.00 | Subsequent myocardial infarction of other sites |
| 96838 | Gyu3400 | [X]Acute transmural myocardial infarction of unspecif site |
| 99991 | Gyu3600 | [X]Subsequent myocardial infarction of unspecified site |

Table S9: Read code list for peripheral valvular disease

| **Pegasus Dictionary Code** | **Read Code** | **Description** |
| --- | --- | --- |
| 996 | G70..11 | Arteriosclerosis |
| 1517 | G73z000 | Intermittent claudication |
| 2065 | G742400 | Embolism and thrombosis of the femoral artery |
| 2760 | G73zz00 | Peripheral vascular disease NOS |
| 3588 | G72z.00 | Aneurysm NOS |
| 3714 | G74z.00 | Arterial embolism and thrombosis NOS |
| 3995 | G70z.00 | Arteriosclerotic vascular disease NOS |
| 4289 | G74..00 | Arterial embolism and thrombosis |
| 4539 | G742500 | Embolism and thrombosis of the popliteal artery |
| 5168 | G70y.00 | Other specified artery atheroma |
| 5640 | G70..00 | Atherosclerosis |
| 5650 | G740.12 | Aortoiliac obstruction |
| 5702 | G73..11 | Peripheral ischaemic vascular disease |
| 5943 | G73..00 | Other peripheral vascular disease |
| 6684 | G723000 | Aneurysm of femoral artery |
| 6827 | G73..13 | Peripheral ischaemia |
| 6853 | G73z011 | Claudication |
| 6900 | G74y500 | Embolism and thrombosis of the subclavian artery |
| 8998 | G74..11 | Arterial embolus and thrombosis |
| 9364 | G74..12 | Thrombosis – arterial |
| 9454 | G72..00 | Other aneurysm |
| 9759 | G718.00 | Leaking abdominal aortic aneurysm |
| 11430 | G715000 | Thoracoabdominal aortic aneurysm, ruptured |
| 13572 | G713.11 | Ruptured abdominal aortic aneurysm |
| 14797 | G702.00 | Extremity artery atheroma |
| 15253 | G740.00 | Embolism and thrombosis of the abdominal aorta |
| 15302 | G742z00 | Peripheral arterial embolism and thrombosis NOS |
| 16068 | G72yz00 | Other aneurysm NOS |
| 16284 | G701.00 | Renal artery atherosclerosis |
| 16366 | G723100 | Aneurysm of popliteal artery |
| 16395 | G722000 | Aneurysm of common iliac artery |
| 16800 | G711.11 | Ruptured thoracic aortic aneurysm |
| 17345 | G714.11 | AAA - Abdominal aortic aneurysm without mention of rupture |
| 17560 | G722.00 | Aneurysm of iliac artery |
| 17767 | G713.00 | Abdominal aortic aneurysm which has ruptured |
| 18478 | G721.00 | Aneurysm of renal artery |
| 19155 | G700.11 | Aorto-iliac disease |
| 23532 | G712.00 | Thoracic aortic aneurysm without mention of rupture |
| 25438 | G720100 | Aneurysm of radial artery |
| 27389 | G72yA00 | Aneurysm of hepatic artery |
| 27494 | G74y300 | Embolism and thrombosis of the iliac artery unspecified |
| 27563 | G711.00 | Thoracic aortic aneurysm which has ruptured |
| 29372 | G742100 | Embolism and thrombosis of the radial artery |
| 30248 | G720200 | Aneurysm of ulnar artery |
| 30495 | G742300 | Embolism and thrombosis of an arm artery NOS |
| 31460 | G74y700 | Embolism and thrombosis of the axillary artery |
| 31876 | G72y000 | Aneurysm of common carotid art |
| 32235 | G74y.00 | Embolism and thrombosis of other specified artery |
| 32634 | G74y100 | Embolism and/or thrombosis of the internal iliac artery |
| 33613 | G720000 | Aneurysm of brachial artery |
| 34159 | G742000 | Embolism and thrombosis of the brachial artery |
| 35529 | G72y400 | Aneurysm of subclavian artery |
| 36390 | G72y200 | Aneurysm of internal carotid artery |
| 38732 | G72y500 | Aneurysm of splenic artery |
| 40787 | G716000 | Thoracoabdominal aortic aneurysm, without mention of rupture |
| 41171 | G72y.00 | Aneurysm of other artery |
| 41597 | G74yz00 | Embolism and thrombosis of other arteries NOS |
| 44085 | G742.00 | Embolism and thrombosis of an arm or leg artery |
| 44835 | G742900 | Embolism and thrombosis of a leg artery NOS |
| 45000 | G723.00 | Aneurysm of leg artery |
| 45645 | G741.00 | Embolism and thrombosis of the thoracic aorta |
| 47655 | G74y800 | Embolism and thrombosis of the coeliac artery |
| 49490 | G74y900 | Embolism and thrombosis of the hepatic artery |
| 50678 | G72y100 | Aneurysm of external carotid artery |
| 52549 | G72y600 | Aneurysm of axillary artery |
| 54865 | G74y000 | Embolism and/or thrombosis of the common iliac artery |
| 56919 | G74y200 | Embolism and/or thrombosis of the external iliac artery |
| 57135 | G72y900 | Aneurysm of inferior mesenteric artery |
| 58698 | G72y300 | Aneurysm of neck artery NOS |
| 58794 | G722200 | Aneurysm of internal iliac artery |
| 59193 | G341200 | Aneurysm of coronary vessels |
| 59492 | G720.00 | Aneurysm of artery of arm |
| 59536 | G72yB00 | Aneurysm of other visceral artery |
| 59538 | G72y800 | Aneurysm of superior mesenteric artery |
| 59671 | G722z00 | Aneurysm of iliac artery NOS |
| 60879 | G722100 | Aneurysm of external iliac artery |
| 62368 | G742200 | Embolism and thrombosis of the ulnar artery |
| 63059 | G723z00 | Aneurysm of leg artery NOS |
| 66823 | G72y700 | Aneurysm of coeliac artery |
| 66981 | G74y600 | Embolism and thrombosis of the splenic artery |
| 67026 | G723200 | Aneurysm of anterior tibial artery |
| 67087 | G341100 | Other cardiac wall aneurysm |
| 69232 | G742600 | Embolism and thrombosis of the anterior tibial artery |
| 69847 | G723300 | Aneurysm of dorsalis pedis artery |
| 71860 | G742700 | Embolism and thrombosis of the dorsalis pedis artery |
| 72062 | G723400 | Aneurysm of posterior tibial artery |
| 94408 | G720z00 | Aneurysm of arm artery NOS |
| 95381 | Gyu7300 | [X]Aneurysm of other specified arteries |
| 99532 | G742800 | Embolism and thrombosis of the posterior tibial artery |
| 100579 | Gyu7000 | [X]Atherosclerosis of other arteries |

Table S10: Read code list for thyroid diseases

| **Pegasus Dictionary Code** | **Read Code** | **Description** |
| --- | --- | --- |
| 273 | C04..13 | Hypothyroidism |
| 1472 | C02..11 | Hyperthyroidism |
| 1658 | R145.00 | [D]Thyroid function test abnormal |
| 1882 | C0...00 | Disorders of thyroid gland |
| 3611 | 1432.00 | H/O: hypothyroidism |
| 3941 | C04z.00 | Hypothyroidism NOS |
| 4937 | 143..11 | H/O: thyroid disorder |
| 6245 | 1431.00 | H/O: hyperthyroidism |
| 10097 | C03..00 | Congenital hypothyroidism |
| 11146 | C134300 | TSH - thyroid-stimulating hormone deficiency |
| 14704 | C04..12 | Thyroid deficiency |
| 18282 | C04z.13 | Hypothyroid goitre, acquired |
| 18598 | 442I.00 | Thyroid function tests abnormal |
| 20970 | 442G.00 | Thyroid hormone tests abnormal |
| 23014 | C04z.12 | Thyroid insufficiency |
| 27278 | 4422.00 | Thyroid hormone tests high |
| 31612 | C03y000 | Congenital hypothyroidism with diffuse goitre |
| 33292 | 4423.00 | Thyroid hormone tests low |
| 34221 | C042.00 | Iodine hypothyroidism |
| 35608 | 1433.00 | H/O: thyroid disorder NOS |
| 35957 | C06z.00 | Thyroid disorder NOS |
| 38976 | C043z00 | Iatrogenic hypothyroidism NOS |
| 48045 | R145z00 | [D]Thyroid function tests abnormal NOS |
| 51481 | C03z.00 | Congenital hypothyroidism NOS |
| 65175 | Cyu1.00 | [X]Disorders of thyroid gland |
| 69290 | C03y.00 | Other specified congenital hypothyroidism |
| 93159 | C03y100 | Congenital hypothyroidism without goitre |
| 93323 | C03z.11 | Congenital thyroid insufficiency |
| 95830 | C047.00 | Subclinical hypothyroidism |
| 102442 | 1JM..00 | Suspected hypothyroidism |
| 106640 | C025.00 | Subclinical hyperthyroidism |
| 108482 | 1JM0.00 | Suspected congenital hypothyroidism |

Table S11: Read code list for chronic obstructive pulmonary disease

| **Pegasus Dictionary Code** | **Read Code** | **Description** |
| --- | --- | --- |
| 148 | H30..00 | Bronchitis unspecified |
| 152 | H302.00 | Wheezy bronchitis |
| 794 | H32..00 | Emphysema |
| 998 | H3...11 | Chronic obstructive airways disease |
| 1001 | H3...00 | Chronic obstructive pulmonary disease |
| 1446 | H312200 | Acute exacerbation of chronic obstructive airways disease |
| 3243 | H31..00 | Chronic bronchitis |
| 3480 | H30z.00 | Bronchitis NOS |
| 5710 | H3z..00 | Chronic obstructive airways disease NOS |
| 5909 | H312011 | Chronic wheezy bronchitis |
| 7092 | H30..12 | Recurrent wheezy bronchitis |
| 7884 | H3y1.00 | Chron obstruct pulmonary dis wth acute exacerbation, unspec |
| 9520 | 66YB.00 | Chronic obstructive pulmonary disease monitoring |
| 9876 | H38..00 | Severe chronic obstructive pulmonary disease |
| 10802 | H37..00 | Moderate chronic obstructive pulmonary disease |
| 10863 | H36..00 | Mild chronic obstructive pulmonary disease |
| 10980 | H322.00 | Centrilobular emphysema |
| 11019 | 8H2R.00 | Admit COPD emergency |
| 11150 | H311.00 | Mucopurulent chronic bronchitis |
| 11287 | 66YM.00 | Chronic obstructive pulmonary disease annual review |
| 12166 | H3y..00 | Other specified chronic obstructive airways disease |
| 14798 | H312100 | Emphysematous bronchitis |
| 15157 | H31z.00 | Chronic bronchitis NOS |
| 15626 | H310000 | Chronic catarrhal bronchitis |
| 16410 | H32yz00 | Other emphysema NOS |
| 17359 | H30..11 | Chest infection - unspecified bronchitis |
| 18207 | H33zz13 | Allergic bronchitis NEC |
| 18476 | 66YL.11 | COPD follow-up |
| 18501 | 66YI.00 | COPD self-management plan given |
| 18621 | 66YL.00 | Chronic obstructive pulmonary disease follow-up |
| 18792 | 9Oi..00 | Chronic obstructive pulmonary disease monitoring |
| 19003 | 66Ye.00 | Emergency COPD admission since last appointment |
| 19106 | 66Yd.00 | COPD accident and emergency attendance since last visit |
| 19428 | 1I70.00 | Chronic obstructive pulmonary disease excluded by spirometry |
| 19434 | 1J71.00 | Suspected chronic obstructive pulmonary disease |
| 21061 | H3y0.00 | Chronic obstruct pulmonary dis with acute lower resp infectn |
| 23492 | H320z00 | Chronic bullous emphysema NOS |
| 24248 | H313.00 | Mixed simple and mucopurulent chronic bronchitis |
| 25603 | H310.00 | Simple chronic bronchitis |
| 26018 | 66YS.00 | Chronic obstructive pulmonary disease monitoring by nurse |
| 26306 | H320.00 | Chronic bullous emphysema |
| 27819 | H312.00 | Obstructive chronic bronchitis |
| 28743 | 66Yf.00 | Number of COPD exacerbations in past year |
| 28755 | 9Oi0.00 | Chronic obstructive pulmonary disease monitoring 1st letter |
| 33450 | H32z.00 | Emphysema NOS |
| 34202 | 9Oi1.00 | Chronic obstructive pulmonary disease monitoring 2nd letter |
| 34215 | 9Oi2.00 | Chronic obstructive pulmonary disease monitoring 3rd letter |
| 37247 | H3z..11 | Chronic obstructive pulmonary disease NOS |
| 37247 | H3z..11 | Chronic obstructive pulmonary disease NOS |
| 37371 | 66YD.00 | Chronic obstructive pulmonary disease monitoring due |
| 37959 | H311100 | Fetid chronic bronchitis |
| 38074 | 9Oi4.00 | Chronic obstructive pulmonary disease monitor phone invite |
| 40159 | H311000 | Purulent chronic bronchitis |
| 40788 | H32y.00 | Other emphysema |
| 42258 | 9Oi3.00 | Chronic obstructive pulmonary disease monitoring verb invite |
| 42313 | 679V.00 | Health education - chronic obstructive pulmonary disease |
| 44525 | H312z00 | Obstructive chronic bronchitis NOS |
| 45770 | 66Yg.00 | Chronic obstructive pulmonary disease disturbs sleep |
| 45771 | 66Yh.00 | Chronic obstructive pulmonary disease does not disturb sleep |
| 45777 | 8CR1.00 | Chronic obstructive pulmonary disease clini management plan |
| 45998 | 66YT.00 | Chronic obstructive pulmonary disease monitoring by doctor |
| 46036 | 66Yi.00 | Multiple COPD emergency hospital admissions |
| 46578 | H321.00 | Panlobular emphysema |
| 46977 | H35z.00 | Allergic alveolitis and pneumonitis NOS |
| 56860 | H320000 | Segmental bullous emphysema |
| 59263 | H32y111 | Acute interstitial emphysema |
| 60188 | H320200 | Giant bullous emphysema |
| 61118 | H310z00 | Simple chronic bronchitis NOS |
| 61513 | H311z00 | Mucopurulent chronic bronchitis NOS |
| 63479 | H32y200 | MacLeod's unilateral emphysema |
| 65733 | Hyu3100 | [X]Other specified chronic obstructive pulmonary disease |
| 66043 | H31y.00 | Other chronic bronchitis |
| 67040 | H3y..11 | Other specified chronic obstructive pulmonary disease |
| 67040 | H3y..11 | Other specified chronic obstructive pulmonary disease |
| 68066 | H31yz00 | Other chronic bronchitis NOS |
| 68662 | H320100 | Zonal bullous emphysema |
| 70787 | H32y100 | Atrophic (senile) emphysema |
| 92955 | H32y000 | Acute vesicular emphysema |
| 93568 | H39..00 | Very severe chronic obstructive pulmonary disease |
| 97800 | 9kf..00 | COPD - enhanced services administration |
| 98284 | 9kf1.00 | Refer COPD structured smoking assessment - enhanc serv admin |
| 99536 | H320300 | Bullous emphysema with collapse |
| 99948 | 9kf0.00 | COPD patient unsuitable for pulmonary rehab - enh serv admin |
| 101042 | 8BMW.00 | Issue of chronic obstructive pulmonary disease rescue pack |
| 102685 | 66YB000 | Chronic obstructive pulmonary disease 3 monthly review |
| 103558 | 8CeD.00 | Preferred place of care for next exacerbation of COPD |
| 103864 | 9kf0.11 | COPD patient unsuitable for pulmonary rehabilitation |
| 103494 | 14B3.12 | History of chronic obstructive pulmonary disease |
| 104481 | 8CMV.00 | Has chronic obstructive pulmonary disease care plan |
| 104710 | 9NgP.11 | On COPD (chr obstruc pulmonary disease) supportv cre pathway |
| 104985 | 9NgP.00 | On chronic obstructive pulmonary disease supprtv cre pathway |
| 104117 | 661M300 | COPD self-management plan agreed |
| 105457 | 8CMW500 | Chronic obstructive pulmonary disease care pathway |
| 106637 | 9Nk7000 | Seen in chronic obstructive pulmonary disease clinic |

Table S12: Read code list for obstructive sleep apnoea

| **Pegasus Dictionary Code** | **Read Code** | **Description** |
| --- | --- | --- |
| 1244 | R005000 | [D]Sleep disturbance, unspecified |
| 2506 | R005311 | [D]Sleep apnoea syndrome |
| 7603 | Fy03.00 | Sleep apnoea |
| 8084 | R005.00 | [D]Sleep disturbances |
| 8148 | Fy03.11 | Obstructive sleep apnoea |
| 20438 | R005312 | [D]Syndrome sleep apnoea |
| 20748 | H5B0.00 | Obstructive sleep apnoea |
| 23779 | H5B..00 | Sleep apnoea |
| 36301 | R005300 | [D]Hypersomnia with sleep apnoea |
| 48539 | R005100 | [D]Insomnia with sleep apnoea |
| 93615 | 9Nk0.00 | Seen in sleep clinic |
| 95887 | 8HTn.00 | Referral to sleep clinic |
| 100177 | 38Da.00 | Berlin questionnaire for sleep apnoea |

Table S13: Prescribed medications in the three months following AF diagnosis stratified by polypharmacy group at baseline and British National Formulary (BNF) Chapters (n=134,745 medications) (Joint Formulary Committee, 2021).

|  | **1-4 medicines**  **(Non- polypharmacy)**  **(n= 21,846 items)** | **5-9 medicines**  **(Polypharmacy)**  **(n=112,899 items)** |
| --- | --- | --- |
| **BNF Chapter 1 (Gastrointestinal system)** | 1,144 (5.2%) | 8,979 (8.0%) |
| **BNF Chapter 2 (Cardiovascular system)** | 15,594 (71.4%) | 67,287 (59.6%) |
| **BNF Chapter 3 (Respiratory system)** | 464 (2.1%) | 4,012 (3.6%) |
| **BNF Chapter 4 (Central nervous system)** | 1,336 (6.1%) | 10,050 (8.9%) |
| **BNF Chapter 5 (Infections)** | 648 (3.0%) | 3,986 (3.5%) |
| **BNF Chapter 6 (Endocrine system)** | 639 (2.9%) | 4,636 (4.1%) |
| **BNF Chapter 7 (Obstetrics, gynaecology and urinary tract disorders)** | 147 (0.7%) | 771 (0.7%) |
| **BNF Chapter 8 (Malignant disease and immunosuppression)** | 48 (0.2%) | 295 (0.3%) |
| **BNF Chapter 9 (Nutrition and blood)** | 334 (1.5%) | 3,013 (2.7%) |
| **BNF Chapter 10 (Musculoskeletal and joint diseases)** | 342 (1.6%) | 2,310 (2.1%) |
| **BNF Chapter 11 (Eye)** | 294 (1.4%) | 2,072 (1.8%) |
| **BNF Chapter 12 (Ear, Nose and Oropharynx)** | 167 (0.8%) | 871 (0.8%) |
| **BNF Chapter 13 (Skin)** | 434 (2.0%) | 2,971 (2.6%) |
| **BNF Chapter 14 (Vaccines)** | 139 (0.6%) | 737 (0.7%) |
| **BNF Chapter 15 (Anaesthesia)** | 11 (0.1%) | 60 (0.1%) |
| **Unable to identify from codes** | 105 (0.5%) | 849 (0.8%) |

Table S14: Prescribed medications in BNF Chapter 2 (Cardiovascular) stratified by polypharmacy group at baseline drug class (n=82,881 medications) (Joint Formulary Committee, 2021).

|  | **1-4 medicines**  **(Non- polypharmacy)**  **(n= 15,594 items)** | **5-9 medicines**  **(Polypharmacy)**  **(n= 67,287** **items)** |
| --- | --- | --- |
| **2.1 Positive Ionotropic Drugs** | 947 (6.1%) | 4,652 (6.9%) |
| **2.2 Diruetics** | 1,134 (7.3%) | 8,696 (12.9%) |
| **2.3 Anti-arrhythmic drugs** | 999 (6.4%) | 2,336 (3.5%) |
| **2.4 Beta-adrenoceptor blocking drugs** | 2,892 (18.5%) | 8,591 (12.8%) |
| **2.5 Hypertension and heart failure drugs** | 1,569 (10.1%) | 10,441 (15.5%) |
| \| **2.6 Nitrates** \| \| --- \| | 1,020 (6.5%) | 6,688 (9.9%) |
| **2.7 Sympathomimetics and other vasoconstrictor drugs** | 0 (0%) | 0 (0%) |
| **2.8 Anticoagulants** | 2,548 (16.3%) | 7,584 (11.3%) |
| **2.9 Antiplatelets** | 3,143 (20.2%) | 10,259 (15.2%) |
| **2.10 Fibrinolytic drugs** | 0 (0%) | 0 (0%) |
| \| **2.11 Antifibrinolytic drugs and haemostatics** \| \| --- \| | 4 (0%) | 7 (0%) |
| **2.12 Lipid regulating drugs** | 1,338 (8.6%) | 8,033 (11.9%) |

Table S15: Prescribed medications in the three months following AF diagnosis stratified by British National Formulary (BNF) Chapters for the propensity score matched groups (n=23,854 medications) (Joint Formulary Committee, 2021).

|  | **1-4 medicines**  **(Matched non- polypharmacy)**  **(n= 7,287 items)** | **5-9 medicines**  **(Matched Polypharmacy)**  **(n=16,567 items)** |
| --- | --- | --- |
| **BNF Chapter 1 (Gastrointestinal system)** | 408 (5.6%) | 1,417 (8.6%) |
| **BNF Chapter 2 (Cardiovascular system)** | 5,149 (70.7%) | 9,714 (58.6%) |
| **BNF Chapter 3 (Respiratory system)** | 137 (1.9%) | 450 (2.7%) |
| **BNF Chapter 4 (Central nervous system)** | 431 (5.9%) | 1,585 (9.6%) |
| **BNF Chapter 5 (Infections)** | 214 (2.9%) | 667 (4.0%) |
| **BNF Chapter 6 (Endocrine system)** | 195 (2.7%) | 497 (3.0%) |
| **BNF Chapter 7 (Obstetrics, gynaecology and urinary tract disorders)** | 60 (0.8%) | 134 (0.8%) |
| **BNF Chapter 8 (Malignant disease and immunosuppression)** | 15 (0.2%) | 35 (0.2%) |
| **BNF Chapter 9 (Nutrition and blood)** | 124 (1.7%) | 476 (2.9%) |
| **BNF Chapter 10 (Musculoskeletal and joint diseases)** | 112 (1.5%) | 393 (2.4%) |
| **BNF Chapter 11 (Eye)** | 121 (1.7%) | 317 (1.9%) |
| **BNF Chapter 12 (Ear, Nose and Oropharynx)** | 59 (0.8%) | 141 (0.9%) |
| **BNF Chapter 13 (Skin)** | 183 (2.5%) | 492 (3.0%) |
| **BNF Chapter 14 (Vaccines)** | 46 (0.6%) | 115 (0.7%) |
| **BNF Chapter 15 (Anaesthesia)** | 4 (0.1%) | 16 (0.1%) |
| **Unable to identify from codes** | 29 (0.4%) | 118 (0.7%) |

**Fig 1: Number of prescribed medications in relation to mortality**


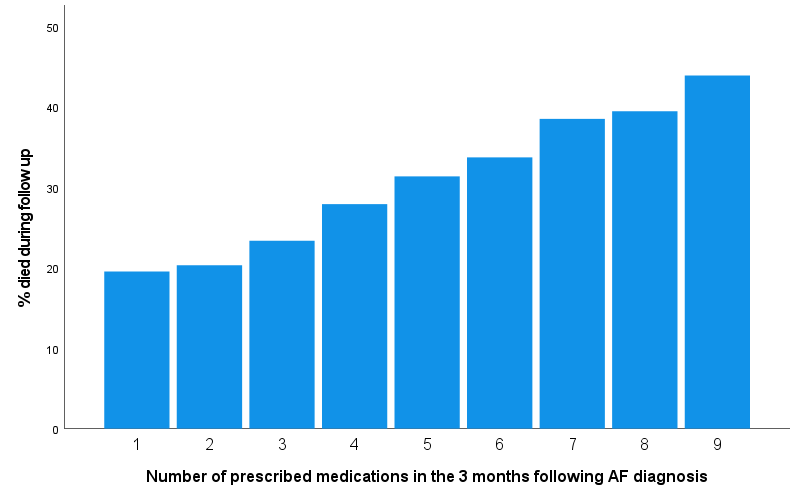

Supplement: Supplementary file 1 — Data S1: [file JOA3-40-47-s001.docx]
